# Phytochemicals That Regulate Neurodegenerative Disease by Targeting Neurotrophins: A Comprehensive Review

**DOI:** 10.1155/2015/814068

**Published:** 2015-05-14

**Authors:** Ramu Venkatesan, Eunhee Ji, Sun Yeou Kim

**Affiliations:** ^1^College of Pharmacy, Gachon University, No. 191, Hambakmoero, Yeonsu-gu, Incheon 406-799, Republic of Korea; ^2^Gachon Medical Research Institute, Gil Medical Center, Inchon 405-760, Republic of Korea; ^3^Gachon Institute of Pharmaceutical Science, Gachon University, No. 191 Hambakmoe-ro, Yeonsu-gu, Incheon 406-799, Republic of Korea

## Abstract

Alzheimer's disease (AD), characterized by progressive dementia and deterioration of cognitive function, is an unsolved social and medical problem. Age, nutrition, and toxins are the most common causes of AD. However, currently no credible treatment is available for AD. Traditional herbs and phytochemicals may delay its onset and slow its progression and also allow recovery by targeting multiple pathological causes by antioxidative, anti-inflammatory, and antiamyloidogenic properties. They also regulate mitochondrial stress, apoptotic factors, free radical scavenging system, and neurotrophic factors. Neurotrophins such as BDNF, NGF, NT3, and NT4/5 play a vital role in neuronal and nonneuronal responses to AD. Neurotrophins depletion accelerates the progression of AD and therefore, replacing such neurotrophins may be a potential treatment for neurodegenerative disease. Here, we review the phytochemicals that mediate the signaling pathways involved in neuroprotection specifically neurotrophin-mediated activation of Trk receptors and members of p75^NTR^ superfamily. We focus on representative phenolic derivatives, iridoid glycosides, terpenoids, alkaloids, and steroidal saponins as regulators of neurotrophin-mediated neuroprotection. Although these phytochemicals have attracted attention owing to their *in vitro* neurotrophin potentiating activity, their *in vivo* and clinical efficacy trials has yet to be established. Therefore, further research is necessary to prove the neuroprotective effects in preclinical models and in humans.

## 1. Introduction

Neurodegenerative diseases are a significant problem. According to a consensus that was developed using the Delphi method, the prevalence of Alzheimer's disease is on the rise, and an estimated 26.6 million patients with AD are reported worldwide. Furthermore, this number is estimated to increase to 106.2 million by 2050 [1]. The global prevalence of Parkinson's disease (PD) is estimated to be 6.3 million patients, with 1.2 million patients in Europe [2]. The frequency of Huntington's disease (HD) was found to be 4–8 in 100000 people in Europe [3], and the prevalence rate of amyotrophic lateral sclerosis (ALS) was determined to be around 2–7 in 100000 people in USA [4]. These neurodegenerative diseases share common symptomological features at different stages of disease progression. The main physiological symptoms of degenerative diseases include elevated oxidative/nitrosative stress, mitochondrial dysfunction, protein misfolding/aggregation, synapse loss, and decreased neuronal survival [5, 6]. When neurons and immune cells are exposed to toxic proteins, a large amount of energy is needed to defend against the accumulated oxygen and nitrogen species that induce stress in the surrounding environment. This results in mitochondrial malfunction with the release of cytochrome C and other mitochondrial proteins, which pave the way towards apoptosis [6]. This overabundance of protein aggregation affects cellular signaling and neuronal function and is a key cause of neuronal loss [7].

AD is recognized as one of the most complicated neurodegenerative diseases, and it is a major social problem. It is a chronic neurodegenerative disorder characterized by progressive dementia and deterioration of cognitive function [8]. As a result of population aging in many countries, the number of people with dementia has been growing rapidly. In addition to elderly patients, dementia can also occur in overweight children. Currently, there is no reliable therapy established for AD. However, recently some convincing evidence has been published regarding the use of herbs and phytochemicals to delay the onset of AD, and it has been shown that early, regular usage of phytochemicals and their derivatives can delay the progression of the disease. Many previous studies reported that regular intake of phytochemicals benefited health by improving mental and physical performance, increasing neuronal cell survival, and boosting the antioxidant system. Neurodegenerative diseases are affected by factors such as stimulating nuclear factor (erythroid-derived 2)-like 2 (Nrf2) in the antioxidant system, sirtuin and forkhead box O (FOXO) transcription factors, and chaperones and neurotrophic factors and by inhibiting acetylcholinesterase (AChE) activity [9, 10]. Additionally, advanced research has led to an increase in the consumption of specific plant ingredients and/or phytochemicals to treat incurable diseases such as neurodegenerative disease [11, 12].

Natural phytochemicals may be less toxic than novel synthetic drugs. However, since these traditional herbal medicines were commonly prepared from crude materials, there are many questions concerning their specific medicinal effects and reproducibility, mechanism of action, and the identity of the active ingredients [13]. Therefore, most recent research has focused on the specific components of an active herb rather than on the herb in its entirety. However, a number of active ingredients still need to be identified and characterized with regard to their potential therapeutic effects, particularly their effects on neurodegenerative diseases.

This review focuses on the phytochemicals and their derivatives that are used to target neurodegenerative diseases by regulating neurotrophins. Accumulating evidence indicates that dietary phytochemicals may prevent or reverse neurodegenerative disease by targeting neurotrophins.

Neurotrophins are important for the survival, maintenance, and regeneration of specific neuronal populations in the brain. The neurotrophins that were identified as neuronal survival-promoting proteins in mammals include nerve growth factor (NGF), brain-derived neurotrophic factor (BDNF), neurotrophin-3 (NT-3), and NT-4/5 [14, 15]. A decrease in neurotrophins has been associated with the pathology of several neurodegenerative diseases and their physiological symptoms [16, 17]. Among the neurotrophins, NGF has been studied extensively as a drug target owing to its strong association to neurodegenerative diseases. The next most common targets are antioxidants, anti-inflammatory and antistress factors, and AChE inhibitors. Neurotrophins are considered to be promising targets for neuroprotective agents against degenerative diseases [18]. Neurotrophin administration might be an effective treatment for neurodegenerative diseases. Until now, neurotrophin-based treatments have progressed as far as preclinical trials; although there are difficulties with such clinical trials, phytochemicals from natural sources, as well as synthetic derivatives, have been shown to have potential as a means of controlling neurotrophin levels. In particular, a modulator or enhancer targeting the tropomyosin-related kinase (Trk) receptor could be a valuable candidate to reverse neurotrophin loss [19]. Several neurotrophins cannot cross the blood brain barrier (BBB); however, this problem can be overcomed by replacing them with neurotrophin-mimetic compounds or with compounds that stimulate neurotrophin expression and can penetrate the BBB.

Additionally, compounds with antioxidant and anti-inflammatory activities have the potential to treat neurodegenerative diseases. For example, ladostigil acts as a neuroprotective agent and has been proposed as an effective treatment of AD and PD. Ladostigil regulates amyloid precursor protein (APP) and inhibits cholinesterase, MAO-B, caspase 3 activation, Bad, and Bax. Ladostigil has also been shown to act on the prosurvival molecule Bcl-2 and increase the availability of ACh and monoamine neurotransmitters in neuroblastoma SK-N-SH cells [20]. Previous studies reported that bioactive polypheonols from herbal drugs played crucial role in the amelioration of neurodegenerative disease mediated by oxidative stress [21]. A novel C-glucosylated xanthone in mangiferin from mango extracts shows medicinal effect related with redox potential functionally [22]. Isothiocynate, a glucosinolate precursor, protects mice from methamphetamine-induced neurotoxicity. It has been reversed by NRF2-mediated stimulation of antioxidant enzyme system too [23]. According to Indian traditional ayurveda system, the velvet bean extract effectively manages memory impairment in PD by reducing GSH, DPPH radicals, and ROS content [24]. EGb 761 (quercetin, kaempferol, isorhamnetin, bilobalide, and ginkgolide) of* Ginkgo biloba* possesses antioxidative effect and helps to improve minimental state of AD in clinical studies [25]. Ginsenosides of* Panax ginseng* protect dopaminergic neurons from 1-methyl-4-phenylpyridinium induced oxidative stress with additional effect by promoting neurotrophic factors [26, 27]. Moreover, curcumin activates NRF2 antioxidative system in animals with AD [28].

Interestingly, ladostigil also enhances the expression of neurotrophic factors and continuously induces neuritogenesis. Multitarget treatments that take advantage of the neurotrophin-enhancing effect of ladostigil seem to be valid evidence for phytochemicals in regulating the brain damage [29].

Additionally, phytochemicals can interact with neuroinflammatory mediators [30, 31] and neurotrophins (NGF, BDNF, NT-3, and NT-5) [9, 14, 15]. Maslinic acid, a potential nutraceutical triterpene, protects cells from ROS and NO by promoting HIF-1*α* and VEGF expression [32]. Further methamphetamine-induced inflammatory cytokines were attenuated by (−)-epigallocatechin-3-gallate from* Camellia sinensis* extract [23]. Tocotrienols from the dietary source effectively reduce neuronal cell death by regulating lipoxygenase, COX-2, and Phospholipase A2 and NF-*κ*B level [33]. Evidently curcuminoid usage in routine Indian diet lowers AD prevalence in India; it might attenuate inflammatory damage by inhibiting cytokine production and microglia activation in AD models [25]. ROS/RNS implicated in cellular inflammation with activation of macrophages and other inflammatory mediators were suppressed by EGCG, Curcumin, and Resveratrol [34]. These properties of phytochemicals perceive attention to therapeutic target for neurodegenerative diseases.

Our group has already reported on natural compounds and their effects on neurotrophin induction. We have previously reported the neurotrophic effects in C6 cells, PC12 cells, and primary astrocytes of (−)-3,5-dicaffeoyl-muco-quinic acid extracted from* Aster scaber,* spicatoside A and the butanol fraction of* Liriope platyphylla, *Gongjin-dan multiherbal traditional medicine,* Cistanches herba* extract, furostanol saponins, diosgenin, diosniponol C-D and diosniposide A-B, and DA-9801 from* Dioscorea spp., *6-shogaol from* Zingiber officinale, *and lignans from* Abies holophylla*. Additionally, these compounds were shown to directly or indirectly function as NGF mimetics or NGF inducers [14, 18, 35–43]. Our research has focused on the use of potential neurotrophin-inducing agents to develop new drugs to combat increasingly prevalent neurodegenerative diseases. We have also conducted an extremely innovative study to create and test new NGF mimetics as a noninvasive treatment for AD.

Overall, phytochemicals provide an effective way of halting neurodegenerative disease. Phytochemicals and derivatives such as 3,7-dihydroxy-2,4,6-trimethoxy-phenanthrene, diosniposide B, lignan derivatives, ginkgolide B, 4,6-dimethoxyphenanthrene-2,3,7-triol, spicatoside A, ginsenoside Rg3, limonoid derivatives, quercetin, cyanidin-3-*O*-*β*-glucopyranoside, clerodane diterpenoids, apigenin derivatives, and quinic acid derivatives induce neuronal cell differentiation and upregulate neurotrophic factors such as NGF and BDNF [18, 35, 44, 45]. These compounds may have the potential to prevent and arrest neurodegeneration by inducing neurotrophic factors and by boosting the activity of certain components of the antioxidant system, such as superoxide dismutase (SOD) and catalase [43]. They may also inhibit the production of reactive oxygen species (ROS) and inflammatory mediators such as nitric oxide (NO), tumor necrosis factor alpha (TNF-*α*), nuclear factor kappa B (NF-*κ*B), interleukin (IL)-1*β*, intrinsic nitric oxide synthase (iNOS), and prostaglandin (PG)E_2_. NGF triggers the TrkA signaling pathway [18, 35, 44, 45] by inhibiting caspase protein expression [46] and via degradation of beta amyloid oligomers in the brain [47]. Phytochemicals such as ladostigil therefore have multiple targets on neurons and appear to be effective in treating neurodegenerative diseases. In particular, polyphenols activate neurotrophins and have antioxidative and antiapoptotic activities in neurons.

Our review focuses on phytochemicals that have the potential to treat neurodegenerative diseases by targeting neurotrophins.

## 2. Cellular and Molecular Interactions That Affect Cognitive Function

### 2.1. Neurotrophins and Receptors

**Figure 1:**
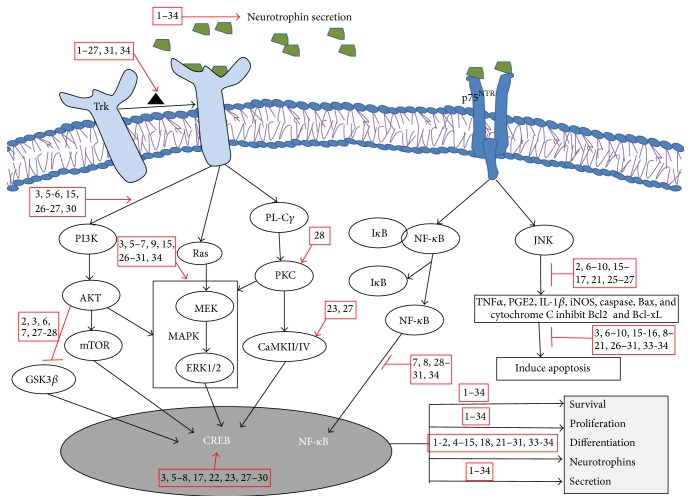
Schematic representation of phytochemicals involved with neurotrophins. By binding to the Trk receptor, neurotrophin signaling mediates cell survival, proliferation, and differentiation through the Ras/MAPK, PI3K/AKT, and PL-C*γ* pathways. NGF-p75^NTR^ receptor binding activates bidirectional cell survival and apoptosis via the NF-*κ*B and JNK pathways, as well as external stress stimuli-mediated generation of ROS with suppression of antioxidative enzyme levels. Trk, tropomyosin-related kinase; p75^NTR^, p75 neurotrophin receptor; NGF, nerve growth factor; BDNF, brain derived neurotrophic factor; LPS, lipopolysaccharide; NADPH, nicotinamide adenine dinucleotide phosphate; PI3K, phosphatidylinsoitol-3-kinase; mTOR, mammalian target of rapamycin; MEK, mitogen-activated protein kinase; MAPK, mitogen activated protein kinase; ERK, extracellular signal-regulated kinases; PL-C*γ*, phospholipase C*γ*; PKC, protein kinase C; NF-*κ*B, nuclear factor-kappa B; JNK, c-Jun N-terminal kinase; I*κ*B, inhibitory kappa B; CREB, cyclic adenosine monophosphate response element binding protein; GSK3*β*, glucose synthase kinase-3*β*; CaMKII/IV, Ca^2+^-calmodulin kinase II/IV. 1: Diosniposide B, 2: Diosgenin, 3: Cyanidin-3-glucopyranoside, 4: 3,7-dihydroxy-2,4,6-trimethoxy-phenanthrene, 5: Spicatoside A, 6: Quercetin, 7: Apigenin, 8: Ginsenoside Rg3, 9: Rosmarinic acid, 10: Ginkgolide B, 11: Limonoid, 12: 4,6-dimethoxy phenanthrene-2,3,7-triol, 13: Furostanol, 14: Coreajaponins B, 15: Quinic acid, 16: Luteolin-7-*O*-*β*-glucopyranoside, 17: Kaempferol, 18: (−)-4,5-dicaffeoyl quinic acid, 19: (−)-3,5-dicaffeoyl mucoquinic acid, 20: (−)-3,4-dicaffeoyl mucoquinic acid, 21: Ginsenosides, 22: Panaxynol, 23: Nigranoic acid, 24: Clerodane diterpenoids, 25: Ligraminol E4-*O*-*β*-d-xyloside, 26: Geniposidic acid, 27: Epigallocatechin-3-galate, 28: Curcumin, 29: Resveratrol, 30: Berberine, 31: 6-shogaol, 32: Oleuropein, 33: Honokiol and magnolol, 34: Huperzine A.

Neurodegenerative diseases might be treated by regulating neuron proliferation, differentiation, and survival. Phytochemicals that inhibit AChE can regulate intracellular signaling and prevent damage to cognitive function in patients with AD by upregulating neurotransmitters in the synaptic environment [48]. The phenolic, flavonoid, anthocyanin, and carotenoid components of* Garcinia parvifolia* fruit extract possess antioxidant and AChE inhibitory properties [48]. Neurotransmitters that are discharged into the postsynaptic cleft target receptors on pre/postsynaptic cells. Once activated, these receptors facilitate various intracellular signaling mechanisms, which promote both short- and long-lasting cellular responses in developing and mature neurons [49]. In a similar fashion, the cells respond to external stimuli through extracellular receptors embedded in the plasma membrane, and neurotrophins individually activate unique members of the Trk receptor family. For example, TrkA, TrkB, and TrkC show high affinity towards NGF, BDNF, and NT 4/5 and NT-3, respectively (Figure 1) [50]. Many neurotrophic factors such as NGF, BDNF, NT-3, NT 4/5, basic fibroblast growth factor-2, and erythropoietin protect neurons from damage. Therefore, they are able to reverse neurodegeneration by interacting with the Trk receptor and promoting the survival, growth, differentiation, and maintenance of neurons [51]. Among the neurotrophins, NGF was the first growth factor to be identified and has been found to promote neuronal survival and neurite ganglia outgrowth in terrestrial birds by using mouse sarcoma tissue [52]. The binding of neurotrophins to their associated receptors facilitates different intracellular signaling cascades, including the Ras/extracellular signal-regulated kinases (ERK), phospholipase C*γ*, and phosphatidylinositol 3-kinase (PI3K)/AKT pathways [19]. Neurotrophins also activate downstream signaling targets to regulate cell survival and promote synaptic and neurite outgrowth in order to maintain cell volume or to promote recovery from neurodegeneration [53]. Neurotrophins promote transcriptional expression of the Trk receptor via Kruppel-like factor 7, Brn3a, cyclic adenosine monophosphate (cAMP) response element-binding (CREB) protein, c-Jun, and NeuroD [54]. An absence of neurotrophins suppresses Trk receptor expression and may cause cognitive neuronal defects. Interestingly, spicatoside A extracted from* Liriope platyphylla *promotes the secretion of neurotrophic factors in C6 glioma and primary astrocyte cells to enhance long-term potentiation (LTP) [42, 44, 55, 56]. Neurotrophins also show weak affinity towards the p75 neurotrophin receptor (p75^NTR^) owing to structural similarities with the Trk family receptors [57]. Interestingly, p75^NTR^ mediates the cell-death-promoting tumor necrosis factor (TNF) receptor superfamily, which includes factors such as FasL, TNF receptor (TNFR)-I, TNFR-II, CD40, OX40, and TNF. Dimeric neurotrophins interact with p75^NTR^ monomers by forming a disulfide bond with cysteine-rich intracellular repeating domains and inducing a conformational change in the receptor. This change then causes enzymatic activation of an adaptor protein via NF-*κ*B and c-Jun N-terminal kinase (JNK), which facilitates proliferation and survival via Bcl-2, or cell death through caspases [58–60].

Neurotrophin binding triggers the activation of the Trk receptor, causing oligomerization and tyrosine residue transautophosphorylation in the intracellular domain. This leads to activation of an intracellular signaling transduction pathway with activation of Ras/mitogen activated protein kinase (MAPK), which results in CREB-dependent neurotrophin secretion and Bcl-2 expression, which promotes cell survival, proliferation, and differentiation (Figure 1) [61]. Previous studies on neurotrophins have been focused mainly on the field of classical neuroscience. In addition to studies of NGF itself, studies of NGF inducers and NGF mimetics are also on the increase. NGF can promote cell survival and differentiation as well as neurite outgrowth, all of which can improve learning and memory in patients with AD. Furthermore, neurotrophin scarcity plays a significant role in neuropathy [52, 62, 63]. Therefore, the study of phytochemicals that can potentiate neurotrophin is necessary in order to find agents to combat neurodegenerative disease.

Recent studies have revealed that, in cerebral cells, amyloid beta (A*β*) 1–40 and A*β* 1–42 can cause stress-induced upregulation of *β*-site APP-cleaving enzyme 1 (BACE 1), which mediates JNK- and p38 MAPK-induced cerebral amyloid angiopathy and vascular degeneration [64]. Montelukast, a leukotriene receptor antagonist, significantly regulates the expression of neuroinflammatory mediators that are responsible for the downregulation of proapoptotic caspase 3 protein expression. Montelukast also mediates the upregulation of Bcl-2 in A*β* 1–42-affected neuronal cells. Moreover, treatment of this antagonist in mice leads to a significant decrease in the latency period in the Y-maze test, and this is established as a new strategy for treating AD [65].

## 3. Role of Dietary Phytochemicals

The brain is an organ that consumes a lot of energy and uses a major proportion of the nutrients consumed by a person. Therefore, certain diets might improve brain function and, for example, it has been shown that dietary lipids contribute to the function of the brain [66]. Consuming dietary macro and micronutrients derived from different traditional medicines has been shown to enhance cognitive function; such nutrients include resveratrol from* Vitis vinifera* (grape), theobromin, xanthin derivatives from* Theobroma cacao* (cocoa), gallic acid from* Vaccinium spp* (blueberry), and catechin, epigallocatechin, and epigallocatechin gallate from* Camellia sinensis* (tea) [67, 68]. It has also been shown that cyanidin-3-glucopyranoside [69], resveratrol, curcumin [13, 70], and flavonoids such as puerarin, rutin, hesperidin, quercetin, genistein, kaempferol, apigenin, and isoliquiritigenin can partly penetrate the BBB. The properties of these phytochemicals can effectively reverse the age-related decline in cognitive function by inducing the expression of neurotrophins via the Trk signaling pathway in the hippocampus [71]. In addition to their special biological activities, plant phytochemicals mainly act as antioxidants, scavenging stress-induced free radicals in the brain. This, in turn, induces neuronal regeneration, neuroprotection, and neurorescue activities that lead to improved neuronal survival, differentiation, LTP, and memory enhancement [72–74]. Below, we describe the complex interactions that occur between phytochemicals from different plant extracts and neurotrophins and expand on the underlying mechanistic signaling pathways and effects that are shown in Table 1.

### 3.1. Neuroprotective Effect of Steroid Phytochemicals

#### 3.1.1. Diosgenin

**Figure 2:**
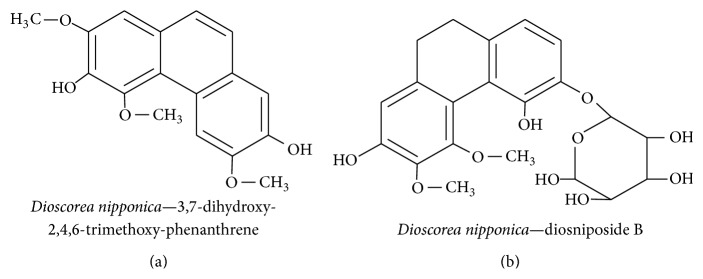


Diosgenin is a constituent of* Dioscorea nipponica* and is widely used as a traditional medicinal plant in Korea to treat diabetes, inflammation, and neurodegenerative diseases. An ethanol extract of* D. nipponica* contained 17 fractions that were investigated to ascertain their effect on NGF secretion in a C6 glioma cell line. The compounds included 3,7-dihydroxy-2,4,6-trimethoxy-phenanthrene (Figure 2(a)) and diosniposide B (Figure 2(b)). Sapogenins in the extract were potent inducers of NGF secretion, were strong reducers of NO production, and were capable of significantly increasing neurite outgrowth in the N2a cell line [18]. It has also been shown that diosgenin can induce NGF expression in a mouse model of diabetic neuropathy. It resulted in increased nerve conduction velocity with ultrastructural changes and stimulation of neural regeneration [38]. Until now, there has been no consensus regarding whether or not phytochemicals from* D. nipponica* can cross the BBB.

#### 3.1.2. 4,6-Dimethoxyphenanthrene-2,3,7-triol

**Figure 3:**
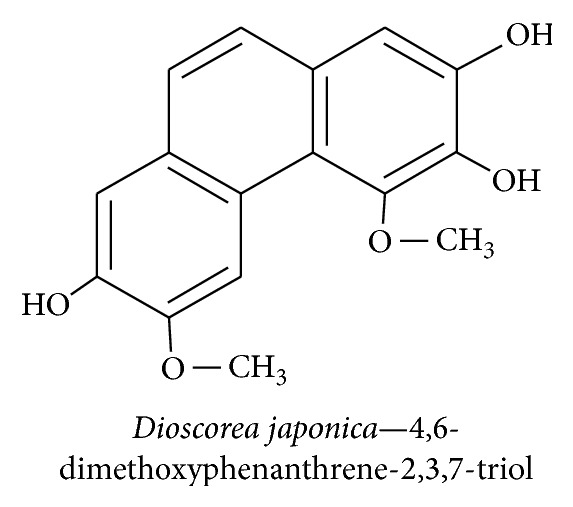


*Dioscorea japonica* is a member of the Dioscoreaceae family and is used as a folk medicine in Korea and China to control hyperglycemia, heart disease, obesity, arthritis, muscular pain, and polyuria disease [39]. We have previously investigated the effects of* D. japonica* extracts isolated from 12 fractions. Two new furostanol saponins, coreajaponins A and B, effectively induced recovery from neurodegenerative disease and diabetic neuropathy. These extracts potentiate NGF secretion and increase neuronal survival and differentiation. We found that coreajaponin A promoted the highest expression of NGF without affecting cell viability [14]. In our latest study, 4,6-dimethoxyphenanthrene-2,3,7-triol (Figure 3) obtained from the extract of* D. japonica* showed NGF agonistic activity in primary Schwann and PC12 cells through TrkA activation, leading to an increase in neurite outgrowth and neuroprotective effect. Thus,* D. japonica* extract has been shown to have beneficial effects and is a potent source of neuroprotective agents, as evidenced by its induction of neurotrophic factors. This effect can also be observed through well-differentiated neurite outgrowth, which has a pivotal role in the treatment of neurodegenerative diseases.

#### 3.1.3. Spicatoside A

**Figure 4:**
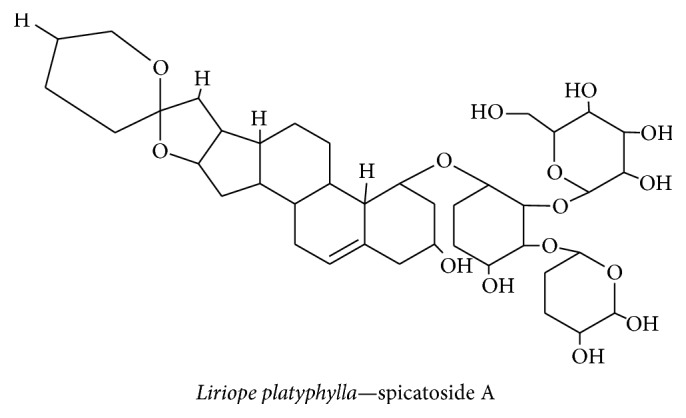


*Liriope platyphylla* is a medicinal plant used in parts of Korea both for its potent action against sortase enzymes in gram-positive bacteria and for its anti-inflammatory effect. The anti-inflammatory property of the* L. platyphylla* extract has attracted considerable attention. In our previous study, we revealed that the* L. platyphylla* extract contains spicatoside A (Figure 4), a steroidal saponin that exerts a neurotrophic effect by inducing neurite outgrowth in PC12 cells and by inducing NGF synthesis in astrocytes through TrkA receptor-mediated PI3-kinase and ERK1/2 activation of CREB, which regulates neuronal function and LTP [35]. A recent study showed that spicatoside A, derived from the extract of* L. platyphylla*, can upregulate the mRNA levels of BDNF in mice and facilitate recovery from cognitive impairment [44]. Therefore, spicatoside A regulates NGF and BDNF secretion—the two major neurotrophins that help to maintain neuronal survival and play functionally active roles in the central nervous system of patients with neurodegenerative diseases.

### 3.2. Neuroprotective Effect of Phenolic Phytochemicals

#### 3.2.1. Quercetin

**Figure 5:**
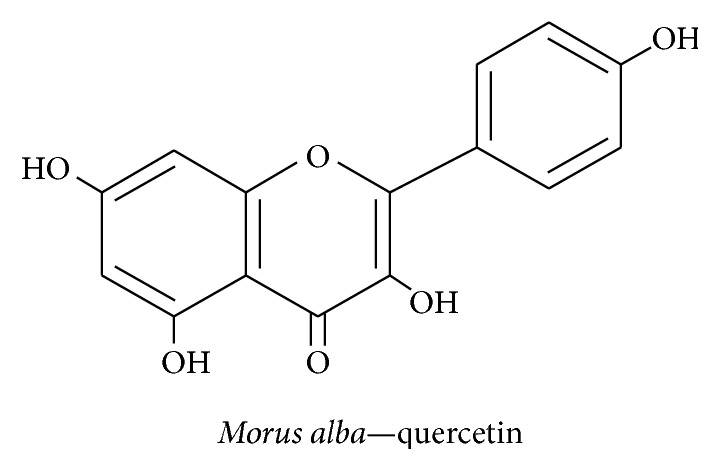


The mulberry fruit* Morus alba* belongs to the Moraceae family, is grown worldwide, and is used to prepare desserts, juice, wine, and vinegar. This fruit contains nutrients such as linoleic acid, palmitic acid, oleic acid, vitamin C, minerals, phenolics, gallic acid, quercetin (Figure 5), and anthocyanins that have antiaging, antioxidant, anti-inflammatory, and anticarcinogenic properties [85, 86]. Cyanidin-3-*O*-*β*-glucopyranoside, a member of the anthocyanin family, easily diffuses across the BBB; its use has been shown to undo the effect of ethanol-induced damage to neurite outgrowth by hampering glycogen synthase kinase-3*β* (GSK-3*β*) in the PI3K pathway in N2a neuroblastoma cell lines [45]. Furthermore, it has been shown that* M. alba* extract can induce NGF secretion via PI3K-mediated ERK1/2 and CREB activation in the mouse hippocampus [69]. Quercetin, a representative flavonoid from* M. alba*, scavenges free radicals through their antioxidant property and can enhance neuroprotection [94]. Thus, quercetin helps promote activity that regulates the neuronal survival rate in the hippocampus.

#### 3.2.2. Apigenin Derivatives

**Figure 6:**
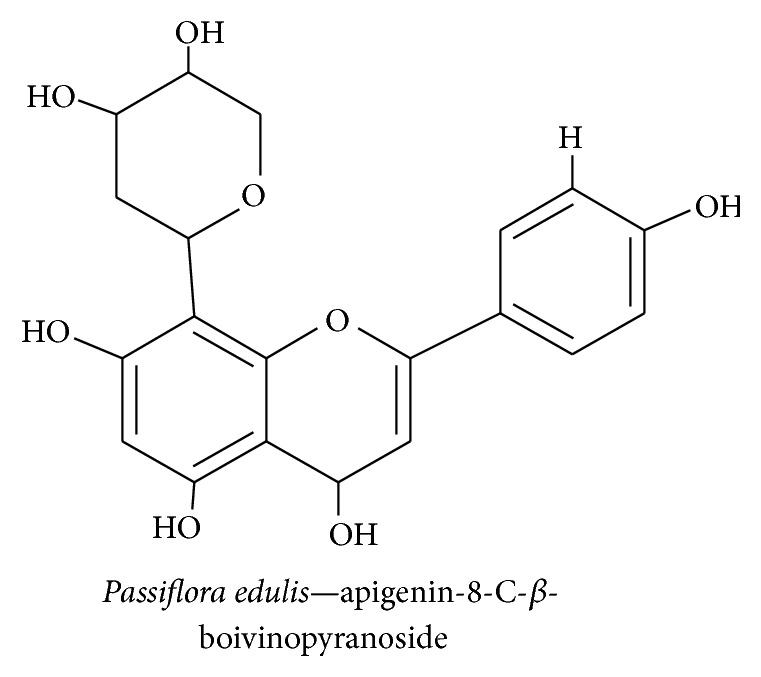


*Passiflora*, commonly named “maracuja,” has been used as a sedative and tranquilizer in Brazilian folk medicine and as a natural anxiolytic agent. Flavonoids from* Passiflora edulis* and* P. alata *have been shown to improve behavioral performance in rats [95]. The phytochemicals that contribute most to the effects of* Passiflora *are flavonoids such as apigenin-8-*C*-*β*-digitoxopyranoside, apigenin-8-*C*-*β*-boivinopyranoside (Figure 6), and luteolin-8-*C*-*β*-boivinopyranoside [88]. Apigenin and its derivatives are known to have anticarcinogenic, antioxidant, and anti-inflammatory properties [96]. Subchronic treatment with apigenin in APP/PS1 mice model downregulates BACE, *β*-CTF, and *β*-amyloid deposition and restores BDNF expression leading to increased memory and synaptic plasticity by ERK1/2/CREB-mediated prevention of AD [97]. Recently, we studied apigenin and found that it also plays a vital role in neurodegenerative disease. It exerts its anti-inflammatory effect on LPS-activated microglia and inhibits NO and PGE_2_ production by scavenging free radicals. Moreover, apigenin suppresses ERK1/2, p38 MAPK, and JNK and modulates NGF-induced neurite outgrowth in PC12 cells [88]. Additionally, apigenin has an apparent permeability coefficient in the BBB, and thus it serves as an effective phytochemical for the treatment of neurodegenerative diseases [98].

#### 3.2.3. Rosmarinic Acid

**Figure 7:**
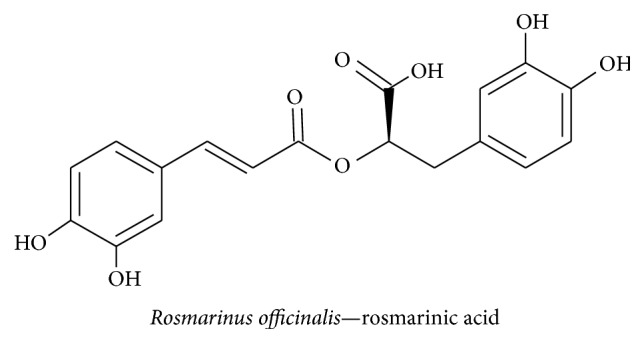


Lemon balm, the common name for* Melissa officinalis*, has been used traditionally for its antioxidant and neuroprotective actions. For example, rosmarinic acid has been shown to scavenge free radicals and prevent apoptosis [99]. Rosmarinic acid (Figure 7) from* Rosmarinus officinalis* exhibits a mimetic neurotrophic effect in PC12 cells by inducing ERK1/2-mediated cell differentiation and enhancing cholinergic activity [100]. Previous studies have demonstrated that* M. officinalis* contains rosmarinic, ursolic, and oleanolic acids, which increases the number of cells and promotes the differentiation of neuroblasts in the dentate gyrus by modulating serum gamma-aminobutyric acid (GABA) transaminase and corticosterone levels [83]. A recent study has revealed that lemon balm oil extracts contain citronellal, geraniol, geranyl acetate, isogeranial, *ε*-caryophyllene, caryophyllene oxide, germacrene D, and carvacrol [101]. These compounds protect against neuronal damage from hypoxia-induced proinflammatory cytokines such as IL-1*β* and TNF-*α* and caspase 3 activity by suppressing hypoxia inducible factor-1*α* (HIF-1*α*) expression [102]. Taken together, these studies show that rosmarinic acid from* M. officinalis *and its derivatives play a vital role in the mechanisms that underlie memory enhancing function by improving cholinergic activity.

#### 3.2.4. Quinic Acid Derivatives

**Figure 8:**
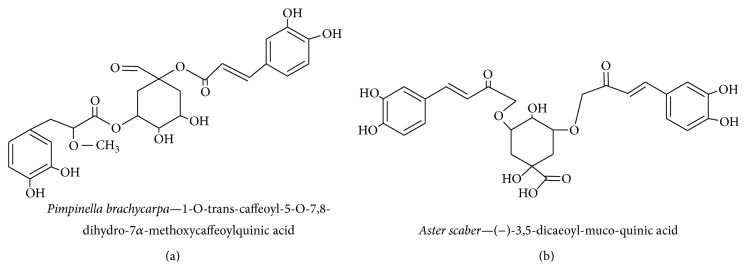


*Pimpinella brachycarpa,* a member of the Apiaceae family, is a source of caffeoylquinic acid and is widely found in Asia, Europe, and Africa; it is used as a folk medicine in Korean culture to treat gastrointestinal tract upsets, asthma, insomnia, and coughs [103].* Aster scaber* is a member of the Asteraceae family that contains (−)-4,5-dicaffeoylquinic acid and (−)-3,5-dicaffeoylmucoquinic acid; it is distributed across eastern Asia, particularly in Korea, and used to treat bruises, headaches, and dizziness [43].* Dipsacus asper*, of the Dipsacaceae family, contains 3,4-dicaffeoylmucoquinic acid and has been used in traditional Chinese medicine for its anti-inflammatory effects on lower back pain, knee pain, rheumatic arthritis, traumatic hematoma, abortion risk, and bone fractures [104]. In our previous study, quinic acid derivatives exhibited antiapoptotic properties against tetrahydropapaveroline- (THP-) treated C6 glioma cells by scavenging free radicals [52]. Fifteen quinic acid derivatives were isolated from* P. brachycarpa *extract, of which 1-*O*-trans-caffeoyl-5-*O*-7,8-dihydro-7*α*-methoxycaffeoylquinic acid (Figure 8(a)) and 3,5-*O*-trans-dicaffeoylquinic acid methyl ester were shown to significantly inhibit NO-mediated iNOS inhibition in LPS-activated BV-2 microglial cell lines [105]. Thus,* P. brachycarpa* extract protects cells by inhibiting NO-mediated neuronal cell loss in BV-2 cells via its antioxidant properties.

Earlier studies have revealed that* A. scaber* extract contains four different quinic acid derivatives, of which (−)-4,5-dicaffeoyl quinic acid and (−)-3,5-dicaffeoyl-muco-quinic acid facilitate neurite outgrowth by protecting against A*β*-induced toxicity in PC12 cells [43, 106]. In a later study on quinic acid isolated from* A. scaber* extract, it was found that quinic acid increased C6 glioma cell survivability upon induction of THP toxicity because of greater malondialdehyde (MDA) and SOD scavenging of free radicals [52]. A further study showed that (−)-3,5-dicaffeoylmucoquinic acid (Figure 8(b)) (from* A. scaber* extract) affects ERK1/2 and PI3K via TrkA signaling cascade activation and subsequently affects neurite outgrowth [43, 106]. Thus, quinic acid from* A. scaber* extract ameliorates neurodegenerative diseases by enhancing the free radical scavenging system and protecting neurons from free radicals and potentiating neurite outgrowth by acting as an NGF mimetic.

*D. asper* extract was shown to potentially act as an antioxidant—with an effectiveness comparable to vitamin E—and protects against aluminum chloride toxicity by protecting cells and reducing A*β* expression in the hippocampus [104]. Hydrogen peroxide- induced toxicity in the SH-SY5Y human neuroblastoma cell line is reduced by (−)-3,5-dicaffeoylmucoquinic acid and (−)-3,4-dicaffeoyl-muco-quinic acid (derived from* D. asper*) owing to an increase in SOD and catalase activity. Thus, quinic acid derivatives prevent oxidative stress in neurodegenerative diseases [107]. Quinic acid derivatives of* D. asper *extract protect neuronal cells from THP-induced cytotoxicity and MDA- and SOD-induced neuronal cells [52]. Compounds extracted from* D. asper* inhibit free radical-induced toxicity by boosting the antioxidant enzyme system.

#### 3.2.5. Epigallocatechin-3-galate

**Figure 9:**
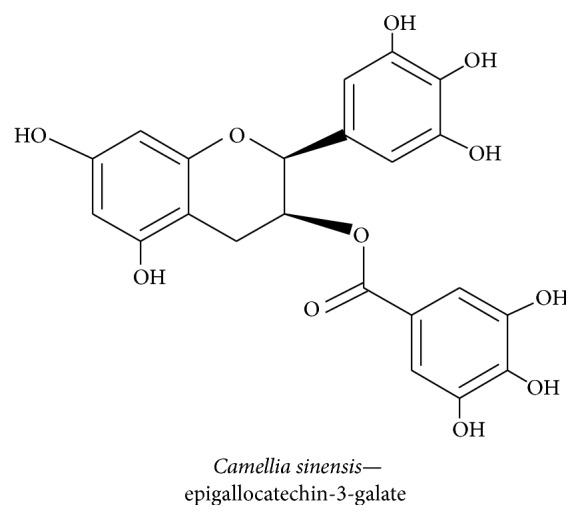


Epigallocatechin-3-galate (EGCG) is a natural green tea polyphenol (GTPP) that is extracted from* Camellia sinensis*, a member of the Theaceae family, that is planted in hilly areas of Asia, in particular China and India, and used as a beverage worldwide [108]. EGCG (Figure 9) ameliorates cognitive impairments in APP/PS1 mice by increasing the expression level of NGF and promoting CREB expression by TrkA phosphorylation through c-Raf/ERK1/2-mediated neuroprotection. EGCG reduces p75/CD and JNK2 activation and cleaved caspase 3 expression which leads to reduced levels of A*β* (1–40) and APP expression in the hippocampus [76]. EGCG potentiates neurite outgrowth in PC12 cells by maintaining the ROS at sublethal level, which was more effective than TrkA/ERK1/2-mediated neurite outgrowth [109]. EGCG reduced hydrogen peroxide-induced apoptosis of PC12 cells by inducing neurite outgrowth by averting PI3K/AKT/GSK-3*β*-mediated activation of caspase 3 and PARP cleavage [110]. Prolonged green tea consumption increases glutathione, potentiates the free radical scavenging system, activates CREB and Bcl-2 protein level, and boosts BDNF expression level thus having a positive effect on age-related neurodegeneration [111]. Green tea also contains catechin, and chronic treatment improves spatial learning and memory by increasing PSD95, BDNF, and CaMKII while suppressing A*β* (1–42) level via the PKA/CREB pathway in the hippocampus of learning-and-memory-impaired SAMP8 mice [112].

#### 3.2.6. Curcumin

**Figure 10:**
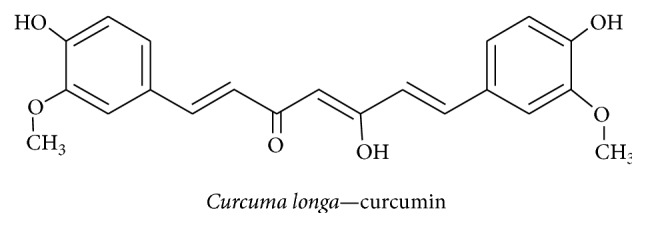


The turmeric plant (*Curcuma longa*) belongs to the ginger family Zingiberaceae and contains the phenolic constituents curcumin, demethoxycurcumin, and bisdemethoxycurcumin. Traditionally, Indian food preparations have been flavored with turmeric since it has good medicinal properties; additionally, it has been used to treat biliary disorders, coughs, diabetic ulcers, hepatic disorders, rheumatism, and sinusitis [113]. Curcumin (Figure 10) promotes PC12 cell neurite outgrowth by inducing a PKC/ERK1/2-mediated increase in CREB expression along with an increase in the expression level of the neurodifferentiation markers GAP43 and NF-L [78]. Recent studies have revealed that curcumin regulates d-galactose induced learning and spatial memory impairment by increasing CREB and BDNF levels in an aged mouse model [114]. Chronic unpredictable stress-induced cognitive deficits in rats were treated with cucurmin; the treatment led to a recovery of BDNF and ERK1/2 levels in the hippocampus [115]. Curcumin affected p53 expression level in cisplatin-treated PC12 cells and reduced the cisplatin-induced inhibition of neurodifferentiation [116]. Curcumin attenuates *β*-amyloid-induced apoptosis by inhibiting NF-*κ*B activation promoted by the p75^NTR^ cell death receptor [117]. Curcumin plays a neuroprotective role in the 6-hydroxydopamine-induced PD rat model by reversing the effect on BDNF level by activating TrkB/PI3K expression and mediating promotion of neural regeneration [118]. Moreover nanoencapsulated curcumin alleviates A*β* (1–42)-induced cognitive impairment in rats by recovering BDNF levels and the AKT/GSK-3 *β* signaling pathway in astrocytes and microglial cells, thus leading to modulation of tau hyperphosphorylation along with an increase in hippocampal synaptophysin levels [119]. Curcumin suppresses the neuroinflammatory mediator TNF-*α* and caspase 3 level by increasing BDNF levels in the olfactory-bulb-ablated rat model [120]. Cucurmin treatment also increased the total level of antioxidants in rats that had been subjected to lead acetate-induced oxidative stress [121]. Therefore, curcumin has been shown to offer neuroprotection and mediate the recovery of learning and memory impairment in various models of neurodegeneration by inducing BDNF and exerting antiapoptotic, antineuroinflammatory, antioxidant, and neuritogenesis-inducing effects.

#### 3.2.7. Resveratrol

**Figure 11:**
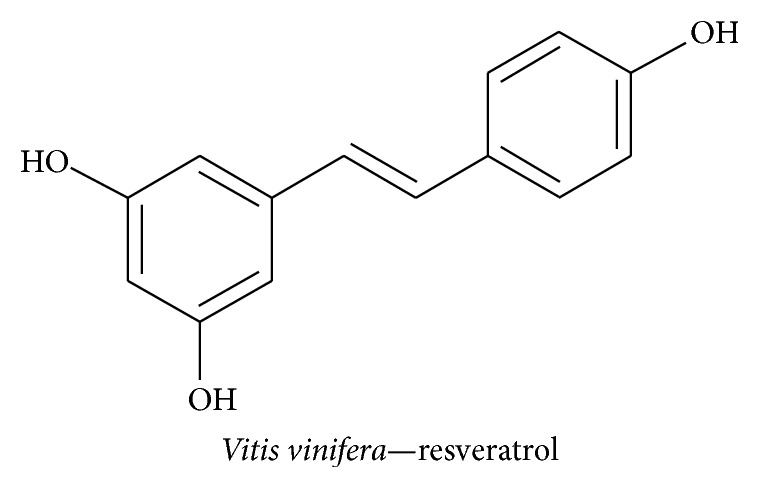


*Vitis vinifera* belongs to the family of Vitaceae and is a rich source of resveratrol (3,5,4′-trihydroxy-trans-stilbene), a polyphenol derivative that has been shown to have antioxidant, anti-inflammatory, phytoestrogenic, vasorelaxant, cardioprotective, and anticarcinogenic activities in the context of neurodegenerative disorders [122]. Resveratrol (Figure 11) prevented chronic cerebral hypofusion in the permanent vessel occlusion rat model by increasing hippocampal NGF, with an effect that persisted for 45 days following surgery, and leading to an improvement in behavioral assessment [92]. Ethanol toxicity-induced apoptosis of Schwann cells could be reversed by resveratrol treatment owing to adenosine monophosphate-activated protein kinase-mediated regulation of BDNF, GDNF, and NGF expression accompanied by inhibition of apoptosis in the peripheral neuronal system [123]. Previous studies revealed that resveratrol attenuates myocardial infract- (MI-) induced MCP-1- and IL-1*β*-mediated inflammation and oxidative stress by modulating SOD and MDA levels and reducing MI-induced NGF level changes [124]. Emotional and spatial cognitive deficits that were induced in rats using chronic unpredictable mild stress were reversed following resveratrol treatment as a result of increases in BDNF level and inhibition of TNF-*α* and IL-1*β* expression levels in the hippocampus; this resulted in a recovery of the latency period of spatial memory [125]. Furthermore, resveratrol suppressed the expression level of the proinflammatory mediator TNF-*α* and the transcription factor NF-*κ*B along with promotion of the anti-inflammatory molecule IL-10 in microglial cells [126]. Additionally resveratrol induces GDNF and BDNF secretion in astrocytes to improve the survival and growth of neurons by significantly activating the ERK1/2/CREB-mediated signaling pathway [127].

#### 3.2.8. Oleuropein

**Figure 12:**
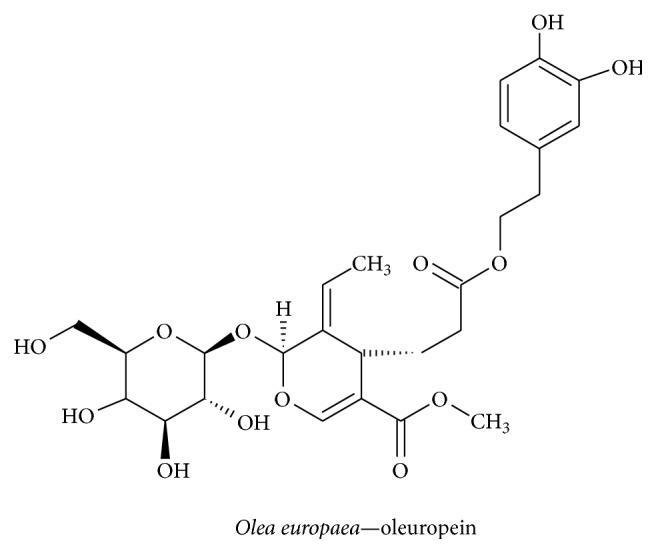


*Olea europaea, *a member of the Oleaceae family, contains the polyphenolic compound oleuropein and is used as a traditional therapy and as a herbal tea; it has been reported to possess hypocholesterolemic, antioxidant, antihypertensive, antiatherogenic, anti-inflammatory, and hypoglycemic properties [128]. Oleuropein (Figure 12) decreased the level of GSH and increased NGF and BDNF levels in serum and elevated the level of NGF in olfactory lobes and the hypothalamus; additionally, it increased BDNF levels in the olfactory lobe but decreased levels of NGF/BDNF in the hippocampus and striatum. Oleuropein did not affect TrkA, TrkB, and P75 expression levels [87]. The antioxidant activity of oleuropein inhibits the aggregation of A*β*42 by reducing the appearance of toxic species in transgenic CL2006 and CL4176 strains of* Caenorhabditis elegans* [129]. Another study revealed that oleuropein aglycon hinders A*β* (1–42) aggregation and eliminates its cytotoxicity [130]. Additionally, the antiamyloidogenic effect of oleuropein was evidenced by a marked elevation of *α*-secretase with a significant reduction in A*β* oligomers in human neuroblastoma SK-N-SH cells [131]. There is evidence that supports phenolic compounds from olive oil crossing the BBB [132].

#### 3.2.9. 6-Shogaol

**Figure 13:**
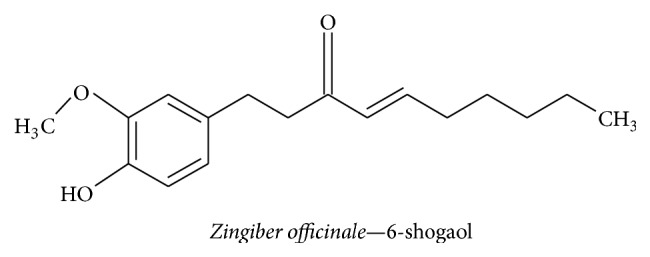


Commonly referred to as ginger,* Zingiber officinale *is a member of the family Zingiberaceae. It contains the compound 6-shogaol, (Figure 13) a phenolic phytochemical, and has been used for centuries as culinary spice and in traditional Indian, Chinese, Arabic, Tibetan, Unani, and Siddha medicinal practices [133]. A wide variety of ginger-derived phytochemicals, including 6-gingerol, 8-gingerol, 10-gingerol, and 6-shogaol, have been shown to have positive effects on nausea, vomiting, and motion sickness [93]. Many studies have shown that 6-shogaol has potent activity against AD, enhances memory, and boosts the antioxidant system [37, 134]. It inhibits inflammatory mediators and improves cognitive function in A*β* (1–42)- and scopolamine-induced dementia mouse models, by increasing the levels of NGF and postsynaptic proteins in the hippocampus [37]. With respect to H_2_O_2_ oxidative stress-induced neuronal apoptosis in astrocytes, 6-shogaol reduces apoptosis by downregulating ROS, Bax, and caspase 3 and upregulating BDNF, GDNF, NGF, Bcl-2, and Bcl-xL via ERK1/2-mediated signaling [135]. In another study using H_2_O_2_-treated HT22 hippocampal neuronal cells, 6-shogaol notably increased choline acetyltransferase, choline transporter, and BDNF expression and reduced ROS production via the BDNF/TrkB-mediated signaling pathway [136]. Additionally, 6-shogaol had a beneficial effect in LPS-treated BV2 and primary microglial cells by inhibiting NO, iNOS, PGE_2_, IL-1*β*, TNF-*α*, Cox-2, P38 MAPK, and NF-*κ*B [137]. Additionally, 6-shogaol provides a neuroprotective effect by attenuating Bax and promoting Bcl-2, Bcl-xL, and BDNF in LPS-treated astrocytes [138]. The studies revealed that 6-shogaol is a valuable phytotherapeutic agent for treating neurodegenerative diseases.

### 3.3. Neuroprotective Effect of Terpenoid-Derived Phytochemicals

#### 3.3.1. Ginkgolide

**Figure 14:**
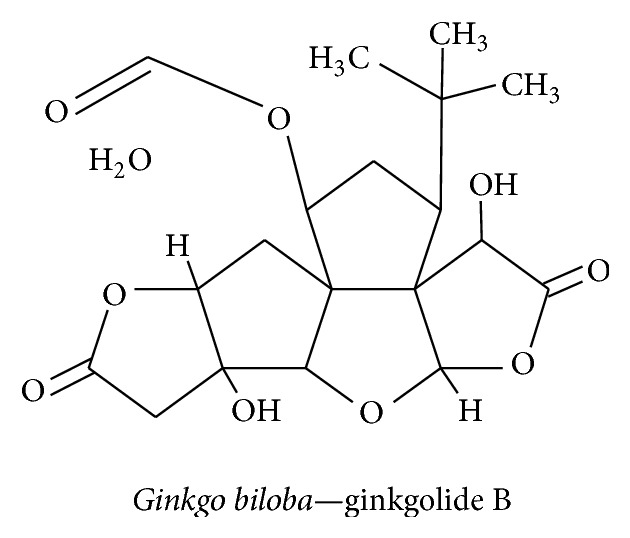


*Ginkgo biloba *belongs to the Ginkgoaceae family. It has been used for thousands of years in traditional Chinese medicine to treat neurological diseases such as neurodegenerative dementia and neurosensory disorders. The active compound in* G. biloba* that is responsible for its neuroprotective action is ginkgolide B (Figure 14).* G. biloba* suppresses ethanol-mediated apoptosis by decreasing the expression of nicotinamide adenine dinucleotide phosphate oxidase and caspase 3 activity in PC12 cells and neurons [80].* G. biloba*'s antioxidant effect attenuates bupivacaine-induced and ROS-dependent mitochondrial and endoplasmic reticulum (ER) dysfunction. This is achieved by suppressing mitochondrial toxicity by reducing the protein levels of cleaved caspase 3 and Htra2 and the protein and mRNA levels of Grp78 and caspase 12 in human neuroblastoma cell lines [139]. In Taiwan, a special kind of dietary supplement named Hu-Yi-neng, which contains* G. biloba* and pine bark extract, has been shown to offer protection against oxidative stress-induced neurodegeneration [140]. Ginkgolide also has the ability to pass through the BBB, especially in ischemic conditions, making it a potent compound for the treatment of neurodegenerative disease [141]. Ginkgolide B has been shown to increase the BDNF expression level in A*β*25–35-treated primary hippocampal neuron cultures by reducing caspase 3, lactate dehydrogenase (LDH), and the K^+^ ion level; it is through this mechanism that it exerts a neuroprotective effect [142].* G. biloba *extract-treatment of rats with streptozotocin-induced type I diabetes significantly increased the expression of NGF and NT-3 in hippocampal neurons [143].* G. biloba *extract has been shown to induce Trk-mediated axonal growth and neuronal protection and suppress apoptotic factors and ROS production.

#### 3.3.2. Limonoids

*Melia toosendan,* of the family Meliaceae, is bitter owing to an abundance of limonoids. These are a family of triterpenoids that function as insecticides, insect antifeedants, and growth regulators in insects. Pharmacologically, limonoids have been shown to have antibacterial, antifungal, antimalarial, anticarcinogenic, antiviral, and neuroprotective effects [84].* M. toosendan* fruit extract has been shown to contain 1*α*,3*α*-dihydroxyl-7*α*-tigloyloxy-12*α*-ethoxylnimbolinin and 12-*O*-ethyl-1-deacetyl-nimbolinin B and induces potent neuronal differentiation by dose-dependently increasing neurite outgrowth in rat pheochromocytoma PC12 cells accompanied by an increased NGF secretion [144].* M. toosendan* extract contains potentially neuroactive compounds that induce neurite outgrowth in a similar manner to NGF and functions through protein kinase A (PKA) and ERK, which decrease during the neuritogenesis of PC12 cells exposed to PKA inhibitors [145].

#### 3.3.3. Ligraminol E4-*O*-*β*-d-xyloside

**Figure 15:**
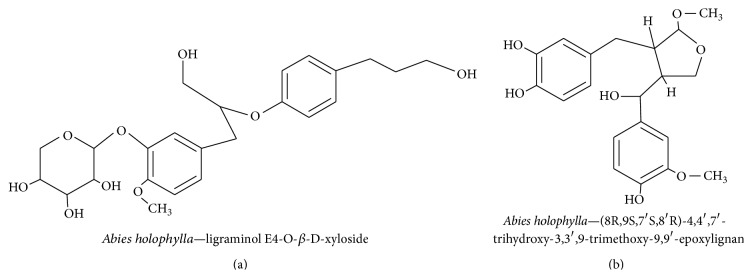


*Abies holophylla* is a member of the Pinaceae family and is commonly known as the Manchurian Fir or the Needle Fir. It is found in evergreen and coniferous forests in Korea, China, and Russia [146]. An ethanol extract of the trunk of* A. holophylla* was found to contain 17 different lignans. Of the 17 lignans, six were new and three were potent inhibitors of LPS-induced NO production in murine microglial cells. In the same study, it was found that three of the isolated lignans increased NGF levels as measured in the supernatants of C6 glial cell cultures [146]. It was found that* A. holophylla* contained two novel sesquiterpenes called ligraminol E4-*O*-*β*-d-xyloside (Figure 15(a)) and (8R,9S,7′S,8′R)-4,4′,7′-trihydroxy-3,3′,9-trimethoxy-9,9′-epoxylignan (Figure 15(b)), which strongly inhibited LPS-activated microglial NO production [75].* Abies spp*. have been shown to potently reduce inflammation and have a protective effect in brain cells.

#### 3.3.4. Clerodane Diterpenoids

**Figure 16:**
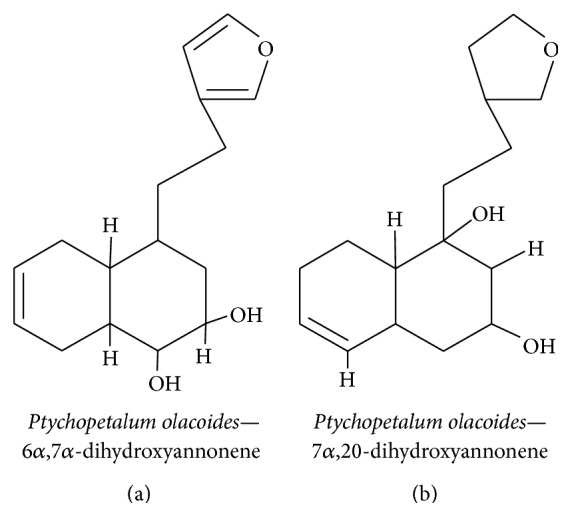


*Ptychopetalum olacoides* is a member of the Olacaceae family and is predominantly found in Brazil. It is commonly used to manage neurodegenerative diseases and other nerve disorders [90]. The methanolic extract of* P. olacoides* contains seven different clerodane-type diterpenoids (Figures 16(a) and 16(b)), two of which promote neurite outgrowth in PC12 cells in a similar manner to NGF [147]. The methanol extract of* P. olacoides* bark contains clerodane diterpenoids with NGF-potentiating activity that induce neurite outgrowth in PC12 cells [90]. Clerodane diterpenenes extracted from* Croton sp.* twigs potentiated NGF-mediated neurite outgrowth in PC12 cells [148]. The compounds acted as NGF mimetics, which supports the use of* P. olacoides* in folk medicines for the treatment of neurological disorders.

#### 3.3.5. Ginsenoside Rg3

**Figure 17:**
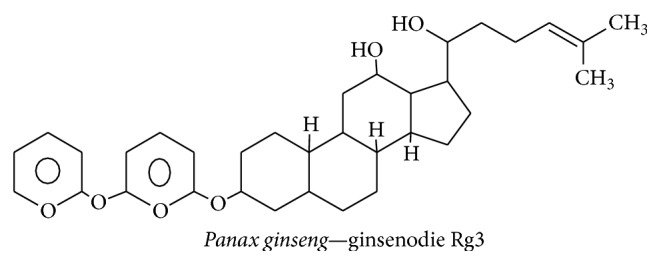


*Panax ginseng*, a member of the family Araliaceae, is a perennial plant grown around Asia—especially in Korea, China, Japan, Russia, and some parts of Vietnam. The main constituents of* P. ginseng* extract are steroidal saponins; the triterpenoid saponin, ginsenoside Rg3, is particularly abundant (Figure 17) [149]. A previous study reported that red ginseng extract was therapeutically effective at controlling inflammatory- and apoptotic-related events associated with neurodegenerative disease. This previous study suggested that red ginseng acts through TNF-*α*, NF-*κ*B, IL-1*β*, and iNOS to reduce A*β*42-induced toxicity on BV-2 cells [89]. Furthermore, it has been shown that ginsenoside Rg3 from* P. ginseng *can increase the phagocytic capacity of canine blood mononuclear cells by stimulating TNF-*α* expression. This induction then leads to the clearing of beta amyloid oligomers in the brain [89, 150].* P. ginseng* extract contains panaxynol, which functions similar to NGF in PC12 cells and induces neuritogenesis through the cAMP and MAPK signaling systems [151]. Moreover, ginsenoside and other derivatives of* P. ginseng *can cross the BBB to an acceptable degree [46]. In addition, the NGF mimetic ginsenoside Rg3 potently enhances cholinergic markers and neuritogenesis via the NGF-TrkA signaling pathway [152]. Therefore,* P. ginseng* extract has the ability to reduce neuroinflammatory cytokines and can induce immune cells to ingest the oligomeric plaques that are formed because of neurodegenerative disease.

#### 3.3.6. 3,4-Secocycloartene Triterpenoid

**Figure 18:**
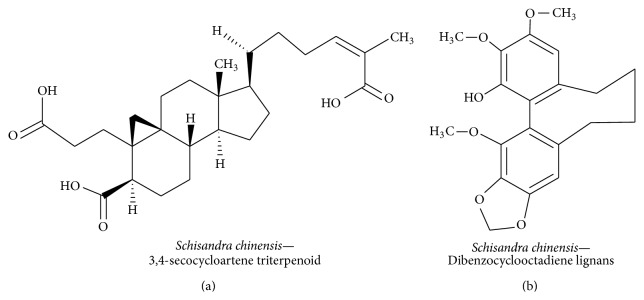


*Schisandra chinensis *belongs to the Schisandraceae family and it has traditionally been used as a refreshment, an antitussive, a tranquillizer, and a treatment for insomnia and fatigue, to increase memory function, and as a sedative [153]. A recent study has shown that the ethyl acetate extract of* S. chinensis* contains 14 different compounds, four of which inhibited LPS-induced NO production in BV-2 murine microglial cells [154]. The compound 3,4-secocycloartene triterpenoid (nigranoic acid) (Figure 18(a)), derived from the* S. chinensis* extract, significantly inhibits NO production and increases BDNF and c-Fos expression in PC12 cells. Moreover, nigranoic acid has been shown to induce neuritogenesis through the Ca^2+^-calmodulin-mediated kinase II and ERK1/2 signaling pathways in PC12 cells [91].* S. chinensis* also contains dibenzocyclooctadiene lignans (Figure 18(b)) and these have been shown to have a potential neuroprotective effect on SH-SY5Y human neuroblastoma cells. Specifically, these isolates have been found to upregulate CREB and Nrf2 via PKA and PKB by inhibiting 6-hydroxydopamine-induced ROS [155].

### 3.4. Alkaloids as Neuroprotectives

#### 3.4.1. Huperzine A

**Figure 19:**
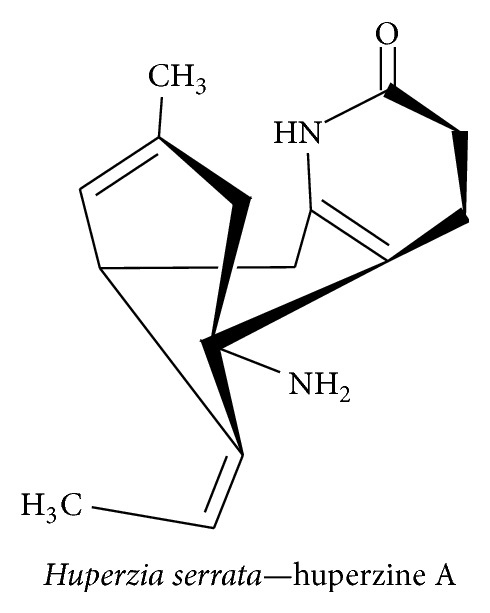


Huperzine A is sesquiterpene alkaloid compound found in* Huperzia serrata* and is a potent reversible acetylcholinesterase (AChE) inhibitor that has been used for many years in Chinese medicine [156]. Huperzine A (Figure 19) exerts a neuroprotective effect against AD by inhibiting AChE, altering A*β* peptide processing, reducing oxidative stress, and promoting the expression of antiapoptotic protein and NGF [81]. The memory deficits in transient cerebral ischemia and reperfusion mouse models were reversed by increasing the expression levels of NGF, BDNF, and TGF-*β* through MAPK/ERK-mediated neuroprotection [157]. In SHSY5Y neuroblastoma cells, huperzine A treatment reversed the reduction in NGF level that was caused by H_2_O_2_-induced oxidative stress; this effect was due to the activation of p75^NTR^ and TrkA receptors and the upstream MAP/ERK signaling pathway [158]. Furthermore, huperzine A promotes neurite outgrowth in rat PC12 cells and in rat cortical astrocyte cells by inhibiting AChE and upregulating the expression levels of NGF and p75^NTR^ [159]. Huperzine A attenuates cognitive defects in streptozotocin-induced diabetic rats by increasing the levels of ChAT, BDNF, SOD, glutathione peroxidase, and catalase while simultaneously inhibiting AChE, MDA, CAT, NF-*κ*B, TNF-*α*, IL-1*β*, IL-6, and caspase-3 [160].

#### 3.4.2. Berberine

**Figure 20:**
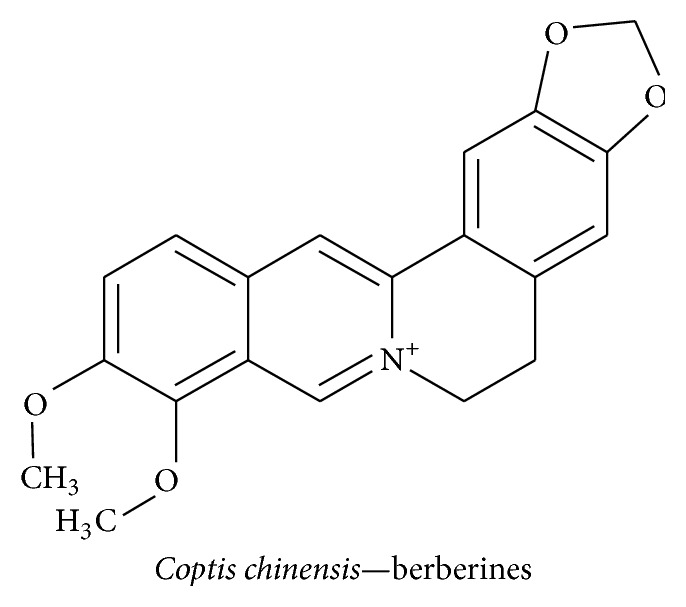


Berberine, an isoquinoline alkaloid, is the major component of* Coptis chinensis*. This plant belongs to the Ranunculaceae family and is used as a herbal medicine to treat skin inflammation, diarrhea, liver disease, and microbial infection in China [161, 162]. Various studies using neurodegenerative disease models have reported that berberine possesses multiple neuroprotective effects including neurotrophin-mediated neuroprotection. It has previously been reported that berberine (Figure 20) attenuates diabetic neuropathy in neuroblastoma cells by inducing hemeoxygenase-1 and NGF expression. ROS levels decreased, and NGF mediated neurite outgrowth increased via the PI3K/Akt/Nrf2-dependent pathway. Similar signaling pathways were shown to play a role in the inhibition of H_2_O_2_-induced neurotoxicity [77]. Berberine isolated from methanol extract of* Coptidis rhizoma* potentiated NGF-induced neurite outgrowth in PC12 cells and inhibited acetylcholinesterase activity as compared to physostigmine [163]. In another study, berberine significantly decreased the expression of the proinflammatory cytokines Cox-2, IL-1*β*, and TNF-*α* and markedly restored levels of BDNF and CREB and reduced the escape latency in rats with scopolamine-induced memory impairments [164]. Berberine pretreatment prevents A*β*-induced IL-6 and MCP-1 production and downregulated Cox-2 and iNOS expression in primary microglia and BV2 cells. This was achieved through activation of AKT/ERK1/2-mediated phosphorylation of I*κ*B-*α* and NF-*κ*B and not stimulation of the JNK pathway [165].

### 3.5. Neuroprotective Effect of Iridoid Glucosides

#### 3.5.1. Geniposidic Acid

*Eucommia ulmoides*, a folk medicine that is traditionally rich in geniposidic acid, is used to strengthen muscles and lungs, control blood pressure and arthritis, avoid miscarriage, improve liver and kidney function, and promote system stamina [166]. A recent study revealed that the bark extract of* E. ulmoides *prevents neurodegenerative diseases such as AD by modulating the regulation of cleaved poly (ADP ribose) polymerase (PARP), cleaved caspase 3, Bcl-2, and Bcl-xL via inhibition of JNK, p38 MAPK, ERK1/2, and PI3K/AKT signaling in H_2_O_2_-treated human SH-SY5Y neuroblastoma cells [79]. A previous study involving mice with scopolamine-induced memory impairments found that* E. ulmoides *bark effectively improved the latency time in the Morris water maze test, increased BDNF expression, and inhibited AChE activity by enhancing cholinergic signaling [167]. Additionally,* E. ulmoides *bark extract protects the brain from A*β* (25–35)-induced impairment of cognitive deficits. This was shown by the reduction in the escape latency in the Morris water maze test following treatment with the extract via inhibition of AChE activity in the hippocampus and frontal cortex [168]. Therefore, the blocking of AChE, matrix metalloproteinases, and cytochrome C release and downregulation of Bcl-2 family proteins, caspase 3 cleavage, and PARP cleavage can all affect mitochondrial structure and function and the neurotransmitter concentration near the affected brain region. This results in protected, functionally active neurons [168].

### 3.6. Neuroprotective Effect of Miscellaneous Phytochemicals

#### 3.6.1. Honokiol and Magnolol

The lignin compounds honokiol and magnolol are isolated from* Magnolia officinalis*—a flower of the Magnoliaceae family that is found in the mountain valleys of China—and possess anticancer, anti-inflammatory, and anxiolytic effects [169]. The two lignin compounds prevent A*β*-induced neuronal cell death by significantly suppressing ROS production, reducing intracellular calcium levels, and inhibiting caspase 3. Additionally, they induce NGF-mediated differentiation of PC12 cells [82]. The novel compound 4-*O*-methylhonokiol promotes ERK1/2-mediated neurite outgrowth in rat embryonic neuronal cells by increasing NGF and BDNF secretion [170]. Furthermore, magnolol enhances BDNF expression levels in the serotonergic system in the brains of rats that have been subjected to unpredictable chronic mild stress [171]. Magnolol also attenuates IL-1*β*, TNF-*α*, IL-6, and ROS production and upregulates AKT and NF-*κ*B levels in the ischemia reperfusion occlusion brain injury model [172]. Additionally, honokiol treatment reversed the neuronal death and dysfunction caused by traumatic brain injury-induced apoptosis by increasing the expression of cell cycle-related proteins, including cyclin D1, CDK4, pRb, and E2F1 [173].

## 4. Conclusion

Based on the above review, several pieces of evidence suggest that naturally occurring phytochemicals that affect neurotrophins and downstream signaling targets should be a first-line treatment of several types of neurodegenerative disease. The review provides a comprehensive discussion of the literature regarding phytochemicals and demonstrates that these compounds offer a safe approach to protect against the neuronal damage caused by neurotrophin deficits and toxin-induced degenerative diseases. Furthermore, they may protect against neuronal loss in patients with neurodegenerative disease. Phytochemicals may be an alternative to other conventional treatment methods. Phytochemicals may control several pathological pathways. In particular, our review stresses the importance of the role of neurotrophins and the value of phytochemicals in regulating neurodegenerative disease. Most previous studies reported similar activities for phytochemicals: (i) reducing oxidative-stress induced free radicals via an antioxidant effect, (ii) boosting the phagocytic properties of immunological cells to aid in the clearance of A*β*/senile plaques in AD, and Lewy bodies in PD, (iii) increasing neurotransmitter concentrations in the vicinity of neurons by inhibiting neurotransmitter cleaving enzymes, (iv) adapting to the prevailing stress conditions by affecting the differentiation properties of neurons, and (v) inhibiting AChE activity. However, phytochemicals that regulate neurodegenerative diseases by the above methods are still underrepresented in preclinical* in vivo* studies. Hence, phytochemicals that regulate neurodegenerative disease by targeting neurotrophins might be a promising future. As nerve growth factor are just responsible for the growth and survival of developing neurons. Phytochemicals that potentiate neurotrophins may not be an absolute cure, but they may serve to prevent or delay the onset of neurodegenerative diseases. Furthermore, based on their chemical structure, phytochemicals do not appear to be cytotoxic. In addition, they provide an appropriate environment for the maintenance of mature neurons and allow neurons to regenerate. As a result, phytochemicals that induce the expression of neurotrophins or mimic neurotrophins and activate Trk receptors can prevent complex and deadly neurodegenerative diseases.

Even though the prevailing view in the field of pharmacology is that it is better to slow or stop neurodegenerative disease progression, a gap still exists between pharmacognosy and pharmacological approaches to the treatment and cure of the disease. In neurodegenerative disease states, the systems, organs, and cells that are under oxidative stress often suffer from unbalanced ionic gradients and are subject to protein interference both inside and outside the signaling pathways. This environment makes the cells, organs, and systems vulnerable to injury. Although dietary phytochemicals with NGF potentiation effects are clearly effective in* in vitro* neuron cell models, many issues need to be addressed before clinical trials. Further studies should be conducted to show the effect of dietary phytochemicals in preclinical* in vivo* models. Particularly, in-depth study is needed to shed more light on which phytochemicals regulate neurodegenerative diseases by regulating NGF-TrkA signaling.

In conclusion, this review highlighted a variety of dietary phytochemicals that affect neurotrophin potential and as such may serve as promising candidates for the treatment of neurodegenerative diseases.

## Figures and Tables

**Table 1:** Plant-derived phytochemicals that affect neurotrophins.

Plant source	Phytochemicals	Pharmacological effects	Medicinal use	Reference
*Aster scaber *	(−)-3,5-Dicaffeoylmucoquinic acid, quinic acid	Activates Trk/ERK1/2/PI3K-mediated neuritogenesis, increases SOD, and reduces MDA activity, neurotrophic mimetic action	Neurodegenerative disease, neuroinflammation, neuritogenesis, and neuroprotection	[52]

*Abies holophylla *	Ligraminol E4-*O*-*β*-d-xyloside, (8R,9S,7′S,8′R)-4,4′,7′-trihydroxy-3,3′,9-trimethoxy-9,9′-epoxylignan, juniperigiside	Inhibits NO production and activates Trk-mediated NGF production	Neurodegenerative disease, neuropathy, neuritogenesis, and neuroinflammatory	[75]

*Camellia sinensis *	Epigallocatechin-3-galate	Activates Trk signaling pathway-mediated neurite outgrowth, PI3K/AKT/GSK-3*β*, induces NGF, BDNF secretion, and inhibits cas3 and ROS level	Neuritogenesis, neuroinflammation, neuroprotection, and cognitive deficit	[76]

*Coptis chinensis *	Berberine	Activates AKT/GSK-3*β*/Nrf2-mediated regulation, cholinergic activity-mediated neurite outgrowth, induces NGF and BDNF secretion, and inhibits Cox2, TNF-*α*, NF-*κ*B, IL-1*β*, and iNOS levels	Neuritogenesis, neuroinflammation, and neuroprotection	[77]

*Curcuma longa *	Curcumin	Activates PKC/ERK-mediated CREB regulation and AKT/GSK-3*β* mediated regulation, induces BDNF secretion, and inhibits Cas3, TNF-*α*, and NF-*κ*B levels	Neuritogenesis, neuroinflammation, and neuroprotection	[78]

*Dioscorea nipponica *	Diosniposide B, 3,7-dihydroxy-2,4,6-trimethoxy-phenanthrene, sapogenin	Activates Trk signaling pathway-mediated neurite outgrowth and induces NGF secretion, inhibits NO	Neuritogenesis, neuroinflammation, and neuroprotection	[18]

*Eucommia ulmoides *	Geniposidic acid	Activates PI3K/AKT, p38 MAPK/ERK1/2-mediated inhibition of LDH, PARP, cleaved caspase 3, MMPs and cytochrome C with increase in Bcl-2, Bcl-xL, BDNF expression, and AChE inhibition	Anti-apoptotic, Alzheimer's disease, neurodegenerative disease, and neuroprotection	[79]

*Ginkgo biloba (L) *	Ginkgolide B	Activates Trk/Ras/MAPK-mediated neurite outgrowth, induces BDNF secretion, and reduces ROS, LDH, caspase3, and proapoptotic factors	Antidepressant, dementia, neuroprotective, nootropic, phosphodiesterase inhibitor, antioxidant, neuritogenesis, and neuroinflammation	[80]

*Huperzia serrata *	Huperzine A	Activates Trk/MAPK/ERK-mediated neurite outgrowth, induces NGF and BDNF secretion, reduces AChE, TNF-*α*, NF-*κ*B, IL-1*β*, and MDA levels, and increases SOD, GSH-Px, Cat, Bcl-2, Bcl-xL, and TGF-*β* level	Neuritogenesis, neuroinflammation, and neuroprotection	[81]

*Liriope platyphylla *	Spicatoside A	Activates Trk, ERK1/2/PI3K-mediated neurite outgrowth and induces NGF and BDNF secretion	Neurodegenerative disease, neuritogenesis, and neuroprotection	[35]

*Magnolia officinalis *	Honokiol, magnolol	Induces NGF and BDNF secretion, inhibits TNF-*α*, NF-*κ*B, IL-1*β*, IL-6, and ROS levels, and increases Akt activity	Neuritogenesis, neuroinflammation, and neuroprotection	[82]

*Melissa officinalis (L) *	Rosmarinic acid, neral/geranial, citronellal, isomenthone, *ε*-caryophyllene, ursolic acid	NGF mimetic, activates ERK1/2-mediated neurite outgrowth, improves cholinergic activity and NF-*κ*B pathway, and inhibits IL-1*β*, TNF-*α*, and caspase 3	Antidepressant, cognitive disorders, neuritogenesis, neuroinflammation, and neuroprotection	[83]

*Melia toosendan *	Limonoid, 1*α*,3*α*-dihydroxyl-7*α*-tigloyloxy-12*α*-ethoxylnimbolinin & 12-*O*-ethyl-1-deacetyl-nimbolinin B	Activates PKA/ERK1/2-mediated neurite outgrowth, induces NGF secretion, and decreases LDH activity	Neurodegenerative disease, neuropathy, neuritogenesis, neuroprotective, and neuroinflammatory	[84]

*Morus alba (L) *	Quercetin, cyanidin-3-*O*-*β*-glucopyranoside, gallic acid	Induces PI3K/ERK1/2-mediated CREB activation, neurite outgrowth, and NGF secretion	Cognitive disorders, antiaging, neuritogenesis, and neuroprotection	[85, 86]

*Olea europaea *	Oleuropein	Induces NGF and BDNF secretion and increases GSH level	Neuroprotection and neuroinflammation	[87]

*Passiflora edulis (L) *	Apigenin-8-*C*-*β*-digitoxopyranoside, apigenin- 8-*C*-*β*-boivinopyranoside, luteolin-8-*C*-*β*-boivinopyranoside	Inhibits NO, iNOS, PGE_2_-mediated modulation of ERK 1/2, p38 MAPK, JNK, and BDNF-induced neurite outgrowth	Neuritogenesis, anxiolytic, neuroinflammation, and neuroprotection	[88]

*Panax ginseng *	Ginsenoside Rg3, panaxynol	Activates cAMP/MAPK & Trk-mediated neuritogenesis, TNF-*α*, NF-*κ*B, IL-1*β*, iNOS, neurotrophic mimetic action	Neuroinflammation, neurodegenerative disease, neuroprotection, and neuritogenesis	[89]

*Pimpinella brachycarpa *	3,5-*O*-*trans*-dicaffeoylquinic acid methyl ester, 1-*O*-*trans*-*p*-coumaroyl-5-*O*-*cis*-*p*-coumaroylquinic acid	Inhibits NO, iNOS production, and boost antioxidant system	Neuroinflammation	[52]

*Ptychopetalum olacoides *	6*α*,7*α*-Dihydroxyannonene, 7*α*,20-dihydroxyannonene, clerodane diterpenoid	Neurotrophic mimetic action and mediates neurite outgrowth	Neurodegenerative disease, neuritogenesis, and neuroprotection	[90]

*Schisandra chinensis *	*α*-Iso-cubebene, dibenzocyclooctadiene lignans, schisanchinins A-D, nigranoic acid	Activates PKA/B/Ca^2+^-CaMKII/ERk1/2-mediated CREB and Nrf2 pathway activation, induces BDNF and c-fos expression, and inhibits NO and PGE_2_ production	Parkinson's disease, neuroinflammation, and neuroprotection	[91]

*Vitis vinifera *	Resveratrol	Activates ERK-mediated CREB regulation, induces NGF, GDNF, and BDNF secretion, and inhibits caspase3, TNF-*α*, NF-*κ*B, IL10, IL-1*β*, MCP1, and MDA levels, increases SOD level.	Neuritogenesis, neuroinflammation, and neuroprotection	[92]

*Zingiber officinale *	6-shogaol	Activates Trk-mediated neurite outgrowth, induces NGF, BDNF, and GDNF secretion, inhibits Cox2, TNF-*α*, NF-*κ*B, IL-1*β*, NO, p38, iNOS, Bax, PG-E2, and ROS level, and increases SOD, Bcl-2, and Bcl-xL levels	Neuritogenesis, neuroinflammation, and neuroprotection	[93]

## References

[1] Brookmeyer R., Johnson E., Ziegler-Graham K., Arrighi H. M. (2007). Forecasting the global burden of Alzheimer's disease. *Alzheimer's & Dementia*.

[2] Rajput A. H. (1992). Frequency and cause of Parkinson's disease. *The Canadian Journal of Neurological Sciences*.

[3] Harper P. S. (1992). The epidemiology of Huntington's disease. *Human Genetics*.

[4] Kurtzke J. F. (1982). Epidemiology of amyotrophic lateral sclerosis. *Advances in Neurology*.

[5] Winner B., Kohl Z., Gage F. H. (2011). Neurodegenerative disease and adult neurogenesis. *European Journal of Neuroscience*.

[6] Finkel T. (2011). Signal transduction by reactive oxygen species. *The Journal of Cell Biology*.

[7] Nakamura T., Lipton S. A. (2007). S-Nitrosylation and uncompetitive/fast off-rate (UFO) drug therapy in neurodegenerative disorders of protein misfolding. *Cell Death & Differentiation*.

[8] Mattson M. P. (2015). Lifelong brain health is a lifelong challenge: from evolutionary principles to empirical evidence. *Ageing Research Reviews*.

[9] Penney J. B., Young A. B. (1981). GABA as the pallidothalamic neurotransmitter: implications for basal ganglia function. *Brain Research*.

[10] Mattson M. P., Son T. G., Camandola S. (2007). Viewpoint: mechanisms of action and therapeutic potential of neurohormetic phytochemicals. *Dose-Response*.

[11] Essa M. M., Vijayan R. K., Castellano-Gonzalez G., Memon M. A., Braidy N., Guillemin G. J. (2012). Neuroprotective effect of natural products against Alzheimer's disease. *Neurochemical Research*.

[12] Howes M.-J. R., Houghton P. J. (2012). Ethnobotanical treatment strategies against alzheimer's disease. *Current Alzheimer Research*.

[13] Kim J., Lee H. J., Lee K. W. (2010). Naturally occurring phytochemicals for the prevention of Alzheimer's disease. *Journal of Neurochemistry*.

[14] Kim K. H., Kim M. A., Moon E. (2011). Furostanol saponins from the rhizomes of *Dioscorea japonica* and their effects on NGF induction. *Bioorganic & Medicinal Chemistry Letters*.

[15] Konar A., Shah N., Singh R. (2011). Protective role of Ashwagandha leaf extract and its component withanone on scopolamine-induced changes in the brain and brain-derived cells. *PLoS ONE*.

[16] Cho T., Ryu J. K., Taghibiglou C. (2013). Long-term potentiation promotes proliferation/survival and neuronal differentiation of neural stem/progenitor cells. *PLoS ONE*.

[17] Fitzsimons C. P., van Bodegraven E., Schouten M. (2014). Epigenetic regulation of adult neural stem cells: implications for Alzheimer’s disease. *Molecular Neurodegeneration*.

[18] Woo K. W., Kwon O. W., Kim S. Y. (2014). Phenolic derivatives from the rhizomes of *Dioscorea nipponica* and their anti-neuroinflammatory and neuroprotective activities. *Journal of Ethnopharmacology*.

[19] Reichardt L. F. (2006). Neurotrophin-regulated signalling pathways. *Philosophical Transactions of the Royal Society B: Biological Sciences*.

[20] Youdim M. B. H., Amit T., Bar-Am O., Weinreb O., Yogev-Falach M. (2006). Implications of co-morbidity for etiology and treatment of neurodegenerative diseases with multifunctional neuroprotective-neurorescue drugs; ladostigil. *Neurotoxicity Research*.

[21] Kumar G. P., Khanum F. (2012). Neuroprotective potential of phytochemicals. *Pharmacognosy Reviews*.

[22] Benard O., Chi Y. (2015). Medicinal properties of mangiferin, structural features, derivative synthesis, pharmacokinetics and biological activities. *Mini-Reviews in Medicinal Chemistry*.

[23] Kita T., Asanuma M., Miyazaki I., Takeshima M. (2014). Protective effects of phytochemical antioxidants against neurotoxin-induced degeneration of dopaminergic neurons. *Journal of Pharmacological Sciences*.

[24] Lampariello L., Cortelazzo A., Guerranti R., Sticozzi C., Valacchi G. (2012). The magic velvet bean of *Mucuna pruriens*. *Journal of Traditional and Complementary Medicine*.

[25] Iriti M., Vitalini S., Fico G., Faoro F. (2010). Neuroprotective herbs and foods from different traditional medicines and diets. *Molecules*.

[26] Chen X.-C., Zhou Y.-C., Fang F., Chen Y., Zhu Y.-G., Chen L.-M. (2005). Ginsenoside Rg1 reduces MPTP-induced substantia nigra neuron loss by suppressing oxidative stress. *Acta Pharmacologica Sinica*.

[27] Rudakewich M., Ba F., Benishin C. G. (2001). Neurotrophic and neuroprotective actions of ginsenosides Rb_1_ and Rg_1_. *Planta Medica*.

[28] Cole G. M., Teter B., Frautschy S. A. (2007). Neuroprotective effects of curcumin. *The Molecular Targets and Therapeutic Uses of Curcumin in Health and Disease*.

[29] Weinreb O., Amit T., Bar-Am O., Youdim M. B. H. (2007). Induction of neurotrophic factors GDNF and BDNF associated with the mechanism of neurorescue action of rasagiline and ladostigil: new insights and implications for therapy. *Annals of the New York Academy of Sciences*.

[30] Verkhratsky A. (2005). Physiology and pathophysiology of the calcium store in the endoplasmic reticulum of neurons. *Physiological Reviews*.

[31] Kwon O. W., Moon E., Chari M. A. (2012). A substituted 3, 4-dihydropyrimidinone derivative (compound D22) prevents inflammation mediated neurotoxicity; role in microglial activation in BV-2 cells. *Bioorganic & Medicinal Chemistry Letters*.

[32] Lozano-Mena G., Sánchez-González M., Juan M., Planas J. (2014). Maslinic acid, a natural phytoalexin-type triterpene from olives—a promising nutraceutical?. *Molecules*.

[33] Frank J., Chin X. W. D., Schrader C., Eckert G. P., Rimbach G. (2012). Do tocotrienols have potential as neuroprotective dietary factors?. *Ageing Research Reviews*.

[34] Darvesh A. S., Carroll R. T., Bishayee A., Geldenhuys W. J., Van der Schyf C. J. (2010). Oxidative stress and Alzheimer's disease: dietary polyphenols as potential therapeutic agents. *Expert Review of Neurotherapeutics*.

[35] Hur J., Lee P., Moon E. (2009). Neurite outgrowth induced by spicatoside A, a steroidal saponin, via the tyrosine kinase A receptor pathway. *European Journal of Pharmacology*.

[36] Moon E., Lee S. O., Kang T. H. (2014). Dioscorea extract (DA-9801) modulates markers of peripheral neuropathy in type 2 diabetic db/db mice. *Biomolecules & Therapeutics*.

[37] Moon M., Kim H. G., Choi J. G. (2014). 6-Shogaol, an active constituent of ginger, attenuates neuroinflammation and cognitive deficits in animal models of dementia. *Biochemical and Biophysical Research Communications*.

[38] Kang T. H., Moon E., Hong B. N. (2011). Diosgenin from *Dioscorea nipponica* ameliorates diabetic neuropathy by inducing nerve growth factor. *Biological and Pharmaceutical Bulletin*.

[39] Kim N., Kim S.-H., Kim Y.-J. (2011). Neurotrophic activity of DA-9801, a mixture extract of *Dioscorea japonica* Thunb. and *Dioscorea nipponica* Makino, in vitro. *Journal of Ethnopharmacology*.

[40] Choi J. G., Moon M., Jeong H. U., Kim M. C., Kim S. Y., Oh M. S. (2011). Cistanches Herba enhances learning and memory by inducing nerve growth factor. *Behavioural Brain Research*.

[41] Moon E., Her Y., Lee J. B. (2009). The multi-herbal medicine Gongjin-dan enhances memory and learning tasks via NGF regulation. *Neuroscience Letters*.

[42] Hur J., Lee P., Kim J., Kim A. J., Kim H., Kim S. Y. (2004). Induction of nerve growth factor by butanol fraction of Liriope platyphylla in C6 and primary astrocyte cells. *Biological and Pharmaceutical Bulletin*.

[43] Hur J. Y., Lee P., Kim H., Kang I., Lee K. R., Kim S. Y. (2004). (−)-3,5-Dicaffeoyl-*muco*-quinic acid isolated from *Aster scaber* contributes to the differentiation of PC12 cells: through tyrosine kinase cascade signaling. *Biochemical and Biophysical Research Communications*.

[44] Kwon G., Lee H. E., Lee D. H. (2014). Spicatoside A enhances memory consolidation through the brain-derived neurotrophic factor in mice. *Neuroscience Letters*.

[45] Chen G., Bower K. A., Xu M. (2009). Cyanidin-3-glucoside reverses ethanol-induced inhibition of neurite outgrowth: role of glycogen synthase kinase 3 beta. *Neurotoxicity Research*.

[46] Wang Z.-J., Nie B.-M., Chen H.-Z., Lu Y. (2006). Panaxynol induces neurite outgrowth in PC12D cells via *c*AMP- and MAP kinase-dependent mechanisms. *Chemico-Biological Interactions*.

[47] Karpagam V., Sathishkumar N., Sathiyamoorthy S. (2013). Identification of BACE1 inhibitors from *Panax ginseng* saponins—an Insilco approach. *Computers in Biology and Medicine*.

[48] Ali Hassan S. H., Fry J. R., Abu Bakar M. F. (2013). Phytochemicals content, antioxidant activity and acetylcholinesterase inhibition properties of indigenous garcinia parvifolia fruit. *BioMed Research International*.

[49] Lovinger D. M. (2010). Neurotransmitter roles in synaptic modulation, plasticity and learning in the dorsal striatum. *Neuropharmacology*.

[50] Barbacid M. (1994). The Trk family of neurotrophin receptors. *Journal of Neurobiology*.

[51] Levi-Montalcini R. (1987). The nerve growth factor: thirty-five years later. *Bioscience Reports*.

[52] Soh Y., Kim J.-A., Sohn N. W., Lee K. R., Kim S. Y. (2003). Protective effects of quinic acid derivatives on tetrahydropapaveroline-induced cell death in C6 glioma cells. *Biological and Pharmaceutical Bulletin*.

[53] Nibuya M., Morinobu S., Duman R. S. (1995). Regulation of BDNF and trkB mRNA in rat brain by chronic electroconvulsive seizure and antidepressant drug treatments. *The Journal of Neuroscience*.

[54] Lei L., Parada L. F. (2007). Transcriptional regulation of Trk family neurotrophin receptors. *Cellular and Molecular Life Sciences*.

[55] Korte M., Carroll P., Wolf E., Brem G., Thoenen H., Bonhoeffer T. (1995). Hippocampal long-term potentiation is impaired in mice lacking brain- derived neurotrophic factor. *Proceedings of the National Academy of Sciences of the United States of America*.

[56] Segal R. A., Greenberg M. E. (1996). Intracellular signaling pathways activated by neurotrophic factors. *Annual Review of Neuroscience*.

[57] Gupta V. K., You Y., Gupta V. B., Klistorner A., Graham S. L. (2013). TrkB receptor signalling: implications in neurodegenerative, psychiatric and proliferative disorders. *International Journal of Molecular Sciences*.

[58] Meakin S. O., Shooter E. M. (1992). The nerve growth factor family of receptors. *Trends in Neurosciences*.

[59] Baud V., Karin M. (2009). Is NF-*κ*B a good target for cancer therapy? Hopes and pitfalls. *Nature Reviews Drug Discovery*.

[60] Hansen K., Wagner B., Hamel W. (2007). Autophagic cell death induced by TrkA receptor activation in human glioblastoma cells. *Journal of Neurochemistry*.

[61] Descamps S., Toillon R.-A., Adriaenssens E. (2001). Nerve growth factor stimulates proliferation and survival of human breast cancer cells through two distinct signaling pathways. *Journal of Biological Chemistry*.

[62] Levi-Montalcini R., Angeletti R. H., Angeletti P. U. (2012). The nerve growth factor. *The Structure and Function of Nervous Tissue V5: Structure III and Physiology III*.

[63] Dauch J. R., Bender D. E., Luna-Wong L. A. (2013). Neurogenic factor-induced Langerhans cell activation in diabetic mice with mechanical allodynia. *Journal of Neuroinflammation*.

[64] Coma M., Guix F. X., Ill-Raga G. (2008). Oxidative stress triggers the amyloidogenic pathway in human vascular smooth muscle cells. *Neurobiology of Aging*.

[65] Lai J., Hu M., Wang H. (2014). Montelukast targeting the cysteinyl leukotriene receptor 1 ameliorates A*β*
_1-42_-induced memory impairment and neuroinflammatory and apoptotic responses in mice. *Neuropharmacology*.

[66] Adibhatla R. M., Hatcher J. F. (2007). Role of lipids in brain injury and diseases. *Future Lipidology*.

[67] Spencer J. P. E. (2009). Flavonoids and brain health: multiple effects underpinned by common mechanisms. *Genes & Nutrition*.

[68] Kuriyama S., Hozawa A., Ohmori K. (2006). Green tea consumption and cognitive function: a cross-sectional study from the Tsurugaya Project. *The American Journal of Clinical Nutrition*.

[69] Kim H. G., Oh M. S. (2013). Memory-enhancing effect of Mori Fructus via induction of nerve growth factor. *British Journal of Nutrition*.

[70] Ramalingam P., Ko Y. T. (2015). Enhanced oral delivery of curcumin from *N*-trimethyl chitosan surface-modified solid lipid nanoparticles: pharmacokinetic and brain distribution evaluations. *Pharmaceutical Research*.

[71] Spencer J. P. E., Vauzour D., Rendeiro C. (2009). Flavonoids and cognition: the molecular mechanisms underlying their behavioural effects. *Archives of Biochemistry and Biophysics*.

[72] Rice-Evans C. A., Miller N. J., Paganga G. (1996). Structure-antioxidant activity relationships of flavonoids and phenolic acids. *Free Radical Biology and Medicine*.

[73] Spencer J. P. E. (2008). Flavonoids: modulators of brain function?. *British Journal of Nutrition*.

[74] Joseph J. A., Shukitt-Hale B., Casadesus G. (2005). Reversing the deleterious effects of aging on neuronal communication and behavior: beneficial properties of fruit polyphenolic compounds. *The American Journal of Clinical Nutrition*.

[75] Xia J.-H., Zhang S.-D., Li Y.-L. (2012). Sesquiterpenoids and triterpenoids from *Abies holophylla* and their bioactivities. *Phytochemistry*.

[76] Liu M., Chen F., Sha L. (2014). (−)-Epigallocatechin-3-gallate ameliorates learning and memory deficits by adjusting the balance of TrkA/p75^NTR^ signaling in *APP/PS1* transgenic mice. *Molecular Neurobiology*.

[77] Hsu Y.-Y., Tseng Y.-T., Lo Y.-C. (2013). Berberine, a natural antidiabetes drug, attenuates glucose neurotoxicity and promotes Nrf2-related neurite outgrowth. *Toxicology and Applied Pharmacology*.

[78] Liao K.-K., Wu M.-J., Chen P.-Y. (2012). Curcuminoids promote neurite outgrowth in PC12 cells through MAPK/ERK- and PKC-dependent pathways. *Journal of Agricultural and Food Chemistry*.

[79] Kwon S.-H., Kim M.-J., Ma S.-X. (2012). *Eucommia ulmoides* Oliv. Bark. protects against hydrogen peroxide-induced neuronal cell death in SH-SY5Y cells. *Journal of Ethnopharmacology*.

[80] Zhang C., Tian X., Luo Y., Meng X. (2011). Ginkgolide B attenuates ethanol-induced neurotoxicity through regulating NADPH oxidases. *Toxicology*.

[81] Zhang H. Y., Tang X. C. (2006). Neuroprotective effects of huperzine A: new therapeutic targets for neurodegenerative disease. *Trends in Pharmacological Sciences*.

[82] Hoi C. P., Ho Y. P., Baum L., Chow A. H. L. (2010). Neuroprotective effect of honokiol and magnolol, compounds from *Magnolia officinalis*, on beta-amyloid-induced toxicity in PC12 cells. *Phytotherapy Research*.

[83] Yoo D. Y., Choi J. H., Kim W. (2011). Effects of *Melissa officinalis* L. (Lemon Balm) extract on neurogenesis associated with serum corticosterone and GABA in the mouse dentate gyrus. *Neurochemical Research*.

[84] Roy A., Saraf S. (2006). Limonoids: overview of significant bioactive triterpenes distributed in plants kingdom. *Biological and Pharmaceutical Bulletin*.

[85] Ercisli S., Orhan E. (2007). Chemical composition of white (*Morus alba*), red (*Morus rubra*) and black (*Morus nigra*) mulberry fruits. *Food Chemistry*.

[86] Peng C.-H., Liu L.-K., Chuang C.-M., Chyau C.-C., Huang C.-N., Wang C.-J. (2011). Mulberry water extracts possess an anti-obesity effect and ability to inhibit hepatic lipogenesis and promote lipolysis. *Journal of Agricultural and Food Chemistry*.

[87] Carito V., Venditti A., Bianco A. (2014). Effects of olive leaf polyphenols on male mouse brain NGF, BDNF and their receptors TrkA, TrkB and p75. *Natural Product Research*.

[88] Xu F., Wang C., Yang L. (2013). *C*-dideoxyhexosyl flavones from the stems and leaves of *Passiflora edulis* Sims. *Food Chemistry*.

[89] Joo S. S., Yoo Y. M., Ahn B. W. (2008). Prevention of inflammation-mediated neurotoxicity by Rg3 and its role in microglial activation. *Biological and Pharmaceutical Bulletin*.

[90] Tang W., Hioki H., Harada K., Kubo M., Fukuyama Y. (2008). Clerodane diterpenoids with NGF-potentiating activity from Ptychopetalum olacoides. *Journal of Natural Products*.

[91] Yuan X.-X., Yang L.-P., Yang Z.-L. (2014). Effect of nigranoic acid on Ca^2+^ influx and its downstream signal mechanism in NGF-differentiated PC12 cells. *Journal of Ethnopharmacology*.

[92] Anastácio J. R., Netto C. A., Castro C. C. (2014). Resveratrol treatment has neuroprotective effects and prevents cognitive impairment after chronic cerebral hypoperfusion. *Neurological Research*.

[93] Palatty P. L., Haniadka R., Valder B., Arora R., Baliga M. S. (2013). Ginger in the prevention of nausea and vomiting: a review. *Critical Reviews in Food Science and Nutrition*.

[94] Kim S. Y., Gao J. J., Lee W.-C., Ryu K. S., Lee K. R., Kim Y. C. (1999). Antioxidative flavonoids from the leaves of *Morus alba*. *Archives of Pharmacal Research*.

[95] Barbosa P. R., Valvassori S. S., Bordignon C. L. (2008). The aqueous extracts of *Passiflora alata* and *Passiflora edulis* reduce anxiety-related behaviors without affecting memory process in rats. *Journal of Medicinal Food*.

[96] Nabavi S. M., Habtemariam S., Daglia M., Nabavi S. F. Apigenin and breast 17 cancers: from chemistry to medicine.

[97] Zhao L., Wang J.-L., Liu R., Li X.-X., Li J.-F., Zhang L. (2013). Neuroprotective, anti-amyloidogenic and neurotrophic effects of apigenin in an Alzheimer's disease mouse model. *Molecules*.

[98] Yang Y., Bai L., Li X. (2014). Transport of active flavonoids, based on cytotoxicity and lipophilicity: an evaluation using the blood-brain barrier cell and Caco-2 cell models. *Toxicology in Vitro*.

[99] Pereira R. P., Fachinetto R., de Souza Prestes A. (2009). Antioxidant effects of different extracts from melissa officinalis, matricaria recutita and cymbopogon citratus. *Neurochemical Research*.

[100] El Omri A., Han J., Yamada P., Kawada K., Abdrabbah M. B., Isoda H. (2010). *Rosmarinus officinalis* polyphenols activate cholinergic activities in PC12 cells through phosphorylation of ERK1/2. *Journal of Ethnopharmacology*.

[101] Nurzyńska-Wierdak R., Bogucka-Kocka A., Szymczak G. (2014). Volatile constituents of *Melissa officinalis* leaves determined by plant age. *Natural Product Communications*.

[102] Bayat M., Azami Tameh A. A., Ghahremani M. H. (2012). Neuroprotective properties of Melissa officinalis after hypoxic-ischemic injury both in vitro and in vivo. *DARU*.

[103] Ahn S., Kim M., Jung I., Sohn H. (2011). Antibacterial, antioxidative and anti-proliferative activity against human colorectal cell of pimpinella brachycarpa. *Korean Journal of Food Preservation*.

[104] Zhang Z.-J., Qian Y.-H., Hu H.-T., Yang J., Yang G.-D. (2003). The herbal medicine *Dipsacus asper Wall* extract reduces the cognitive deficits and overexpression of *β*-amyloid protein induced by aluminum exposure. *Life Sciences*.

[105] Lee S. Y., Moon E., Kim S. Y., Lee K. R. (2013). Quinic acid derivatives from *Pimpinella brachycarpa* exert anti-neuroinflammatory activity in lipopolysaccharide-induced microglia. *Bioorganic & Medicinal Chemistry Letters*.

[106] Xiao H.-B., Cao X., Wang L. (2011). 1,5-Dicaffeoylquinic acid protects primary neurons from amyloid *β* 1-42-induced apoptosis via PI3K/Akt signaling pathway. *Chinese Medical Journal*.

[107] Kim S.-S., Park R.-Y., Jeon H.-J., Kwon Y.-S., Chun W. (2005). Neuroprotective effects of 3,5-dicaffeoylquinic acid on hydrogen peroxide-induced cell death in SH-SY5Y cells. *Phytotherapy Research*.

[108] Gramza-Michalowska A., Regula J. (2007). Use of tea extracts (*Camelia sinensis*) in jelly candies as polyphenols sources in human diet. *Asia Pacific Journal of Clinical Nutrition*.

[109] Gundimeda U., McNeill T. H., Schiffman J. E., Hinton D. R., Gopalakrishna R. (2010). Green tea polyphenols potentiate the action of nerve growth factor to induce neuritogenesis: possible role of reactive oxygen species. *Journal of Neuroscience Research*.

[110] Koh S.-H., Kim S. H., Kwon H. (2003). Epigallocatechin gallate protects nerve growth factor differentiated PC12 cells from oxidative-radical-stress-induced apoptosis through its effect on phosphoinositide 3-kinase/Akt and glycogen synthase kinase-3. *Molecular Brain Research*.

[111] Andrade J. P., Assunção M. (2012). Protective effects of chronic green tea consumption on age-related neurodegeneration. *Current Pharmaceutical Design*.

[112] Li Q., Zhao H. F., Zhang Z. F. (2009). Long-term green tea catechin administration prevents spatial learning and memory impairment in senescence-accelerated mouse prone-8 mice by decreasing A*β*1–42 oligomers and upregulating synaptic plasticity-related proteins in the hippocampus. *Neuroscience*.

[113] Akbik D., Ghadiri M., Chrzanowski W., Rohanizadeh R. (2014). Curcumin as a wound healing agent. *Life Sciences*.

[114] Nam S. M., Choi J. H., Yoo D. Y. (2014). Effects of curcumin (*Curcuma longa*) on learning and spatial memory as well as cell proliferation and neuroblast differentiation in adult and aged mice by upregulating brain-derived neurotrophic factor and CREB signaling. *Journal of Medicinal Food*.

[115] Liu D., Wang Z., Gao Z. (2014). Effects of curcumin on learning and memory deficits, BDNF, and ERK protein expression in rats exposed to chronic unpredictable stress. *Behavioural Brain Research*.

[116] Mendonça L. M., da Silva Machado C., Correia Teixeira C. C., Pedro de Freitas L. A., Pires Bianchi M. D. L., Greggi Antunes L. M. (2013). Curcumin reduces cisplatin-induced neurotoxicity in NGF-differentiated PC12 cells. *NeuroToxicology*.

[117] Kuner P., Schubenel R., Hertel C. (1998). *β*-Amyloid binds to p75(NTR) and activates NF*κ*B in human neuroblastoma cells. *Journal of Neuroscience Research*.

[118] Yang J., Song S., Li J., Liang T. (2014). Neuroprotective effect of curcumin on hippocampal injury in 6-OHDA-induced Parkinson's disease rat. *Pathology Research and Practice*.

[119] Hoppe J. B., Coradini K., Frozza R. L. (2013). Free and nanoencapsulated curcumin suppress *β*-amyloid-induced cognitive impairments in rats: Involvement of BDNF and Akt/GSK-3*β* signaling pathway. *Neurobiology of Learning and Memory*.

[120] Rinwa P., Kumar A., Garg S. (2013). Suppression of neuroinflammatory and apoptotic signaling cascade by curcumin alone and in combination with piperine in rat model of olfactory bulbectomy induced depression. *PLoS ONE*.

[121] Hosseinzadeh S., Roshan V. D., Mahjoub S. (2013). Continuous exercise training and curcumin attenuate changes in brain-derived neurotrophic factor and oxidative stress induced by lead acetate in the hippocampus of male rats. *Pharmaceutical Biology*.

[122] Ma T., Tan M. S., Yu J. T., Tan L. (2014). Resveratrol as a therapeutic agent for Alzheimer’s disease. *BioMed Research International*.

[123] Yuan H., Zhang J., Liu H., Li Z. (2013). The protective effects of resveratrol on Schwann cells with toxicity induced by ethanol in vitro. *Neurochemistry International*.

[124] Xin P., Pan Y., Zhu W., Huang S., Wei M., Chen C. (2010). Favorable effects of resveratrol on sympathetic neural remodeling in rats following myocardial infarction. *European Journal of Pharmacology*.

[125] Yazir Y., Utkan T., Gacar N., Aricioglu F. (2015). Resveratrol exerts anti-inflammatory and neuroprotective effects to prevent memory deficits in rats exposed to chronic unpredictable mild stress. *Physiology & Behavior*.

[126] Song J., Cheon S. Y., Jung W., Lee W. T., Lee J. E. (2014). Resveratrol induces the expression of interleukin-10 and brain-derived neurotrophic factor in BV2 microglia under hypoxia. *International Journal of Molecular Sciences*.

[127] Zhang F., Lu Y.-F., Wu Q., Liu J., Shi J.-S. (2012). Resveratrol promotes neurotrophic factor release from astroglia. *Experimental Biology and Medicine*.

[128] El S. N., Karakaya S. (2009). Olive tree (Olea europaea) leaves: potential beneficial effects on human health. *Nutrition Reviews*.

[129] Diomede L., Rigacci S., Romeo M., Stefani M., Salmona M. (2013). Oleuropein aglycone protects transgenic *C. elegans* strains expressing A*β*42 by reducing plaque load and motor deficit. *PLoS ONE*.

[130] Rigacci S., Guidotti V., Bucciantini M. (2011). A*β*(1–42) aggregates into non-toxic amyloid assemblies in the presence of the natural polyphenol oleuropein aglycon. *Current Alzheimer Research*.

[131] Kostomoiri M., Fragkouli A., Sagnou M. (2013). Oleuropein, an anti-oxidant polyphenol constituent of olive promotes *α*-secretase cleavage of the amyloid precursor protein (A*β*PP). *Cellular and Molecular Neurobiology*.

[132] Serra A., Rubió L., Borràs X., Macià A., Romero M.-P., Motilva M.-J. (2012). Distribution of olive oil phenolic compounds in rat tissues after administration of a phenolic extract from olive cake. *Molecular Nutrition & Food Research*.

[133] Haniadka R., Rajeev A. G., Palatty P. L., Arora R., Baliga M. S. (2012). *Zingiber officinale* (ginger) as an anti-emetic in cancer chemotherapy: a review. *The Journal of Alternative and Complementary Medicine*.

[134] Chen H., Soroka D. N., Hu Y., Chen X., Sang S. (2013). Characterization of thiol-conjugated metabolites of ginger components shogaols in mouse and human urine and modulation of the glutathione levels in cancer cells by [6]-shogaol. *Molecular Nutrition & Food Research*.

[135] Kim S., Kwon J. (2013). [6]-Shogaol attenuates neuronal apoptosis in hydrogen peroxide-treated astrocytes through the up-regulation of neurotrophic factors. *Phytotherapy Research*.

[136] Shim S., Kwon J. (2012). Effects of [6]-shogaol on cholinergic signaling in HT22 cells following neuronal damage induced by hydrogen peroxide. *Food and Chemical Toxicology*.

[137] Ha S. K., Moon E., Ju M. S. (2012). 6-Shogaol, a ginger product, modulates neuroinflammation: a new approach to neuroprotection. *Neuropharmacology*.

[138] Shim S., Kim S., Kwon Y.-B., Kwon J. (2012). Protection by [6]-shogaol against lipopolysaccharide-induced toxicity in murine astrocytes is related to production of brain-derived neurotrophic factor. *Food and Chemical Toxicology*.

[139] Li L., Zhang Q.-G., Lai L.-Y. (2013). Neuroprotective effect of ginkgolide B on bupivacaine-induced apoptosis in SH-SY5Y cells. *Oxidative Medicine and Cellular Longevity*.

[140] Yang Y.-H., Hsieh T.-J., Tsai M.-L., Chen C.-H., Lin H.-T., Wu S.-J. (2014). Neuroprotective effects of Hu-Yi-Neng, a diet supplement, on SH-SY5Y human neuroblastoma cells. *THE Journal of Nutrition, Health & Aging*.

[141] Fang W., Deng Y., Li Y. (2010). Blood brain barrier permeability and therapeutic time window of Ginkgolide B in ischemia-reperfusion injury. *European Journal of Pharmaceutical Sciences*.

[142] Xiao Q., Wang C., Li J. (2010). Ginkgolide B protects hippocampal neurons from apoptosis induced by beta-amyloid 25–35 partly via up-regulation of brain-derived neurotrophic factor. *European Journal of Pharmacology*.

[143] Zhao J., Jin K.-K., Wu L., Chen G.-R., Li J.-M. (2012). Effects of extract of ginkgo biloba on learning and memory ability and NGF and NT-3 expression in diabetic rats. *Zhongguo Ying Yong Sheng Li Xue Za Zhi*.

[144] Zhang Q., Li J.-K., Ge R., Liang J.-Y., Li Q.-S., Min Z.-D. (2013). Novel NGF-potentiating limonoids from the fruits of *Melia toosendan*. *Fitoterapia*.

[145] Yu J. C. H., Min Z.-D., Ip N. Y. (2004). Melia toosendan regulates PC12 cell differentiation via the activation of protein kinase A and extracellular signal-regulated kinases. *NeuroSignals*.

[146] Kim C. S., Kwon O. W., Kim S. Y., Lee K. R. (2013). Bioactive lignans from the Trunk of *Abies holophylla*. *Journal of Natural Products*.

[147] Tang W., Kubo M., Harada K., Hioki H., Fukuyama Y. (2009). Novel NGF-potentiating diterpenoids from a Brazilian medicinal plant, *Ptychopetalum olacoides*. *Bioorganic & Medicinal Chemistry Letters*.

[148] Sun Y., Wang M., Ren Q. (2014). Two novel clerodane diterpenenes with NGF-potentiating activities from the twigs of *Croton yanhuii*. *Fitoterapia*.

[149] Chung H. S., Lee Y.-C., Kyung Rhee Y., Lee S.-Y. (2011). Consumer acceptance of ginseng food products. *Journal of Food Science*.

[150] Kang K. A., Kang J. H., Yang M. P. (2008). Ginseng total saponin enhances the phagocytic capacity of canine peripheral blood phagocytes in vitro. *The American Journal of Chinese Medicine*.

[151] Wang Z.-J., Nie B.-M., Chen H.-Z., Lu Y. (2006). Panaxynol induces neurite outgrowth in PC12D cells via cAMP- and MAP kinase-dependent mechanisms. *Chemico-Biological Interactions*.

[152] Kim M. S., Yu J. M., Kim H. J. (2014). Ginsenoside Re and Rd enhance the expression of cholinergic markers and neuronal differentiation in neuro-2a cells. *Biological and Pharmaceutical Bulletin*.

[153] Zhang C., Zhao X., Mao X. (2014). Pharmacological evaluation of sedative and hypnotic effects of schizandrin through the modification of pentobarbital-induced sleep behaviors in mice. *European Journal of Pharmacology*.

[154] Hu D., Yang Z., Yao X. (2014). Dibenzocyclooctadiene lignans from *Schisandra chinensis* and their inhibitory activity on NO production in lipopolysaccharide-activated microglia cells. *Phytochemistry*.

[155] Park S. Y., Son B. G., Park Y. H., Kim C.-M., Park G., Choi Y.-W. (2014). The neuroprotective effects of *α*-iso-cubebene on dopaminergic cell death: involvement of CREB/Nrf2 signaling. *Neurochemical Research*.

[156] Zhu X. Z., Li X.-Y., Liu J. (2004). Recent pharmacological studies on natural products in China. *European Journal of Pharmacology*.

[157] Wang Z.-F., Tang L.-L., Yan H., Wang Y.-J., Tang X.-C. (2006). Effects of huperzine A on memory deficits and neurotrophic factors production after transient cerebral ischemia and reperfusion in mice. *Pharmacology Biochemistry and Behavior*.

[158] Tang L.-L., Wang R., Tang X.-C. (2005). Huperzine A protects SHSY5Y neuroblastoma cells against oxidative stress damage via nerve growth factor production. *European Journal of Pharmacology*.

[159] Tang L.-L., Wang R., Tang X.-C. (2005). Effects of huperzine A on secretion of nerve growth factor in cultured rat cortical astrocytes and neurite outgrowth in rat PC12 cells. *Acta Pharmacologica Sinica*.

[160] Mao X.-Y., Cao D.-F., Li X. (2014). Huperzine a ameliorates cognitive deficits in streptozotocin-induced diabetic rats. *International Journal of Molecular Sciences*.

[161] Asai M., Iwata N., Yoshikawa A. (2007). Berberine alters the processing of Alzheimer's amyloid precursor protein to decrease A*β* secretion. *Biochemical and Biophysical Research Communications*.

[162] Kim M. H., Kim S., Yang W. M. (2014). Mechanisms of action of phytochemicals from medicinal herbs in the treatment of Alzheimer*ʼ*s disease. *Planta Medica*.

[163] Ishima T., Nishimura T., Iyo M., Hashimoto K. (2008). Potentiation of nerve growth factor-induced neurite outgrowth in PC12 cells by donepezil: role of sigma-1 receptors and IP_3_ receptors. *Progress in Neuro-Psychopharmacology and Biological Psychiatry*.

[164] Lee B., Sur B., Shim I., Lee H., Hahm D.-H. (2012). *Phellodendron amurense* and its major alkaloid compound, berberine ameliorates scopolamine-induced neuronal impairment and memory dysfunction in rats. *The Korean Journal of Physiology and Pharmacology*.

[165] Jia L., Liu J., Song Z. (2012). Berberine suppresses amyloid-beta-induced inflammatory response in microglia by inhibiting nuclear factor-kappaB and mitogen-activated protein kinase signalling pathways. *Journal of Pharmacy and Pharmacology*.

[166] Jiang L., Wang Z., Zhu H.-W. (2011). Beneficial effect of *Eucommia* polysaccharides on systemic lupus erythematosus-like syndrome induced by *Campylobacter jejuni* in BALB/c mice. *Inflammation*.

[167] Kwon S.-H., Ma S.-X., Joo H.-J., Lee S.-Y., Jang C.-G. (2013). Inhibitory effects of Eucommia ulmoides Oliv. Bark on scopolamine-induced learning and memory deficits in mice. *Biomolecules and Therapeutics*.

[168] Kwon S.-H., Lee H.-K., Kim J.-A. (2011). Neuroprotective effects of *Eucommia ulmoides* Oliv. Bark on amyloid beta25-35-induced learning and memory impairments in mice. *Neuroscience Letters*.

[169] Lee Y. K., Song J. K., Choi I. S. (2010). Neurotrophic activity of obovatol on the cultured embryonic rat neuronal cells by increase of neurotrophin release through activation of ERK pathway. *European Journal of Pharmacology*.

[170] Lee Y. K., Choi I. S., Kim Y. H. (2009). Neurite outgrowth effect of 4-O-methylhonokiol by induction of neurotrophic factors through ERK activation. *Neurochemical Research*.

[171] Li L.-F., Lu J., Li X.-M. (2012). Antidepressant-like effect of magnolol on BDNF up-regulation and serotonergic system activity in unpredictable chronic mild stress treated rats. *Phytotherapy Research*.

[172] Chen J. H., Kuo H. C., Lee K. F., Tsai T. H. (2014). Magnolol protects neurons against ischemia injury via the downregulation of p38/MAPK, CHOP and nitrotyrosine. *Toxicology and Applied Pharmacology*.

[173] Wang H., Liao Z., Sun X. (2014). Intravenous administration of Honokiol provides neuroprotection and improves functional recovery after traumatic brain injury through cell cycle inhibition. *Neuropharmacology*.

